# The effect of surface ligands on the surface chemical states and photoluminescence characteristics in cesium lead bromide perovskite nanocrystals

**DOI:** 10.1039/d5ra05099e

**Published:** 2025-08-28

**Authors:** Muhammad Asharuddin, Rahmat Hidayat, Adhita Asma Nurunnizar, Natalita Maulani Nursam, Valdi Rizki Yandri, Waode Sukmawati Arsyad, Joko Suwardy, Efi Dwi Indari, Yoshiyuki Yamashita

**Affiliations:** a Physics of Magnetism and Photonics Research Division, Faculty of Mathematics and Natural Sciences, Bandung Institute of Technology Jl. Ganesha 10 Bandung 40132 West Java Indonesia rahmat@itb.ac.id; b Research Center for Electronics, National Research and Innovation Agency (BRIN), KST Samaun Samadikun Jl. Sangkuriang Bandung 40135 West Java Indonesia; c Department of Electrical Engineering, Polytechnic State of Padang Limau Manis Padang 25164 West Sumatra Indonesia; d Physics Department, Faculty of Mathematics and Natural Sciences, Halu Oleo University Anduonohu Kendari South East Sulawesi 93232 Indonesia; e Research Center for Quantum Physics, National Research and Innovation Agency (BRIN) KST BJ Habibie Serpong Banten 15314 Indonesia; f Nano Electronics Device Materials Group, Research Center for Electronic and Optical Materials, National Institute for Materials Science (NIMS) 305-0044 1-1 Namiki Tsukuba Ibaraki Japan yamashita.yoshiyuki@nims.go.jp; g Graduate School of Engineering, Kyushu University Motooka 744, Nishi-ku Fukuoka 819-0395 Japan

## Abstract

This paper presents the results of our study on the relationship between the surface chemical states, which are influenced by ligands, and photoluminescence (PL) characteristics in cesium lead halide perovskite nanocrystals (NCs). NCs were synthesized *via* the Ligand-Assisted Reprecipitation (LARP) and Ultrasonic-Assisted (URSOA) methods, which were able to produce NCs with and without ligands. Although both synthesis methods used similar precursor composition and processing steps, the resulting crystal structures of NCs are different. The LARP method yielded orthorhombic CsPbBr_3_ NCs, while the URSOA method yielded a mixture of hexagonal Cs_4_PbBr_6_ NCs and orthorhombic CsPbBr_3_ NCs with an approximate weight ratio of ∼10 : 1. The X-ray diffraction data indicated that both NCs with and without ligands have the same crystal structure. However, photoelectron spectroscopy (XPS and HAXPES) analysis showed that chemical states in NCs without ligands differ between the inner side and the surface, which could be associated with surface defect species from the accumulation of Cs^+^ atoms, Pb atoms with zero oxidation state (Pb^0^), unbonded Br atoms, and Br vacancies at the surface of the NCs. The difference appears to be correlated with the observed PL characteristics. Although photoelectron spectroscopy measures the core level orbitals, the measured chemical states may indicate electronic structure alteration in valence orbitals, which are involved in photoexcitation and exciton relaxation processes. The PL of LARP NCs (orthorhombic CsPbBr_3_) shows two components of PL decay, which are largely suppressed in NCs with purification or NCs without ligands. However, for URSOA NCs (predominantly Cs_4_PbBr_6_ NCs), the PL decays are almost similar for both with and without ligands. The present experimental results clearly show that the variations in PL characteristics, besides the crystal structure that determines the intrinsic properties of the formed excitons, may also come from surface states or surface defect species influenced by surface ligands. In addition, the results can also explain the much higher degree of defect tolerance properties in URSOA NCs compared to LARP NCs. The insights gained from this work may be useful not only for further development of passivation molecules in a general context but also for designing buffer layer molecules in perovskite heterojunction devices.

## Abstract 2

This paper presents the results of our study on the relationship between the surface chemical states, which are influenced by ligands, and photoluminescence (PL) characteristics in cesium lead halide perovskite nanocrystals (NCs).

## Graphical abstract

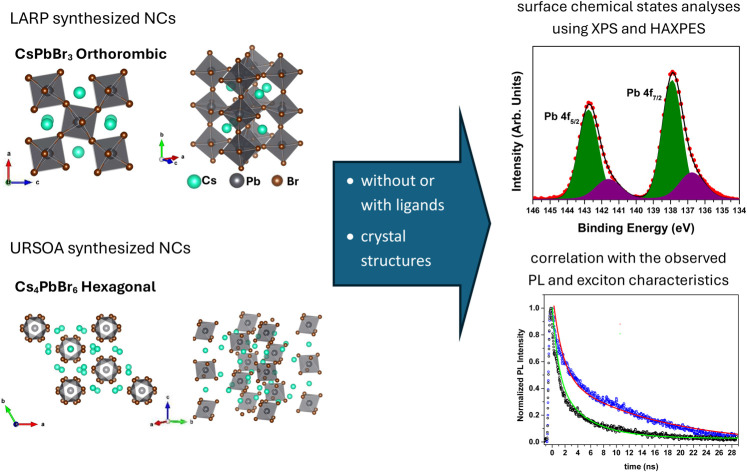

## 1. Introduction

Lead-halide perovskite nanocrystals (NCs) constitute a class of emerging materials of significant interest because of the possibility of tailoring their electronic and optical properties, which results in modifications of their bandgap energy, emission wavelength, and charge-carrier mobility.^1–4^ Therefore, lead halide perovskite NCs have been much explored as promising nanomaterials for application in optoelectronic devices such as solar cells,^5^ photodetectors,^6^ lasers,^7^ and light-emitting devices.^8^ Lead-halide perovskite materials can typically be classified into two categories based on the type of their cations, *i.e.*, organic lead halide perovskites (often also referred as hybrid lead halide perovskites) and all inorganic lead halide perovskites. Organic lead halide perovskites incorporate organic monocation, such as methylammonium (MA^+^/CH_3_NH_3_^+^), formamidinium (FA^+^/CH_5_N_2_^+^), or other similar molecules.^9^ Other halide anions, particularly bromide (Br^−^), have also been widely reported, which showed a larger open circuit voltage and better chemical stability despite a smaller power conversion efficiency compared to those with iodide (I^−^).^10^ In contrast, instead of incorporating organic molecular cations, inorganic lead halide perovskites contain inorganic cations of alkali metals, the first group of the periodic table, such as rubidium (Rb)^11^ and cesium (Cs).^12^ Compared to organic lead halide perovskites, inorganic cesium lead halides exhibit better long-term stability under ambient conditions.^13^

Lead-halide perovskite NCs are usually synthesized by wet chemical processes, where various shapes of NCs can be obtained such as quantum dots (QDs), nanoplates, and nanowires.^14^ Various methods, such as hot injection,^15^ solvent-induced reprecipitation,^16^ microwave-assisted (MA),^17^ ultrasonic-assisted (URSOA),^18^ and ligand-assisted reprecipitation (LARP) methods, have been developed to synthesize these perovskite NCs with relatively high products reproducibility and homogeneity.^19,20^ The LARP method is the most widely utilized method because of high reproducibility with the possibility of varying ligands, precursors and solvents.^21^ During the synthesis process, the ligands play important roles in dissolving the precursors, crystal seed formation, controlling crystal growth, and crystal surface encapsulation by the ligands.^22,23^ In LARP, however, the crystal seed formation must be initiated by the addition of an antisolvent to the precursor solution.^24^ Lead-halide perovskite QDs with high photoluminescence quantum yields are commonly synthesized using this LARP method.^25^ In addition, by choosing appropriate ligands and their composition ratios, the synthesis can produce NCs with a particular crystal structure, such as monoclinic, orthorhombic,^26^ tetragonal^27^ or cubic structures.^27^ Another synthesis method under consideration is the URSOA method, which also uses similar precursors and ligands as used in the LARP method. However, the URSOA method employs ultrasonic wave, which plays important roles to initiate the nucleation of crystal seed and control the crystal growth.^18,28,29^ This method does not require the heating of precursors and could be implemented as a “one pot” synthesis process. Molecular vibrations produced by ultrasonic waves cause local heating inside the precursor solution, allowing a rapid formation of NCs in an entirely room temperature environment.

The photoluminescence (PL) characteristics and stability of these lead halide perovskites have recently become a major focus of research. The PL characteristics of perovskite NCs have been assigned to be originated from exciton with its various intrinsic properties, such as free-excitons, self-trapped excitons (STEs), and exciton polarons.^30–32^ The PL characteristics are also determined by crystal structure. CsPbBr_3_, one of the most investigated lead halide perovskites, shows different PL wavelength and intensity depending on the crystal structure, namely orthorhombic, tetragonal, or cubic structures.^26,27^ Various synthesis parameters, such as precursor concentrations, ligands, antisolvent polarity, *etc.*, might significantly affect the PL characteristics of the synthesis products despite the products showing the same crystal structure.^30^ Moreover, although the synthesized lead-halide QDs or NCs show a perfect crystal structure, from the X-ray diffraction (XRD) measurement for instance, and can effectively absorb photons, some samples may show weak PL, suggesting the occurrence of strong non-radiative recombination pathways.^33,34^ This non-radiative sites perhaps not present inside the NCs, but on the NCs surface.^35,36^ It is therefore important to understand the relationship between the PL characteristics and the surface chemical states, which may be influenced by surface ligands, to verify the cause of the observation of PL characteristic variation. However, only a limited number of studies have been reported in detail on the effect of ligands on the PL characteristics, the crystal structures of the NCs, and the surface chemical states at the NC surface.^13,33^ Here, we report our study on the effects of ligands on the crystal structures of the cesium lead bromide perovskite NCs, the chemical states at the surfaces, and the PL characteristics by performing several characterizations using X-ray diffraction (XRD), X-ray photoelectron spectroscopy (XPS), hard X-ray photoelectron spectroscopy (HAXPES), and Fourier transform infrared spectroscopy (FTIR). This work establishes a direct correlation between surface states and PL characteristics that has not been explicitly addressed in previous studies. Among the aforementioned synthesis methods, in this study, we chose the LARP and URSOA methods for synthesizing cesium lead bromides because both LARP and URSOA can use the same composition and concentrations of precursors and ligands. In addition, those methods can produce both NCs with and without ligands, which are useful for achieving the aim of the present study.^18,27,37^

## 2. Experimental section

### 2.1. Materials

Cesium bromide (CsBr, > 99.99%) and lead bromide (PbBr_2_, < 99,99%), purchased from Tokyo Chemical Industry Co., Ltd, were used as the perovskite precursors. Linoleic acid (LA) and Oleylamine (OlAm) (purchased from Tokyo Chemical Industry Co., Ltd) were used as ligands. Dimethyl sulfoxide (DMSO) and dimethylformamide (DMF) were used as precursor solvents to synthesize cesium lead bromide NCs. Toluene was used as the antisolvent, and ethanol was used as the solvent for the purification process. Analytical-grade solvents (Merck/Sigma Aldrich) were used without further purification.

### 2.2. Synthesis of perovskite NCs

#### 2.2.1 LARP method

**Fig. 1:**
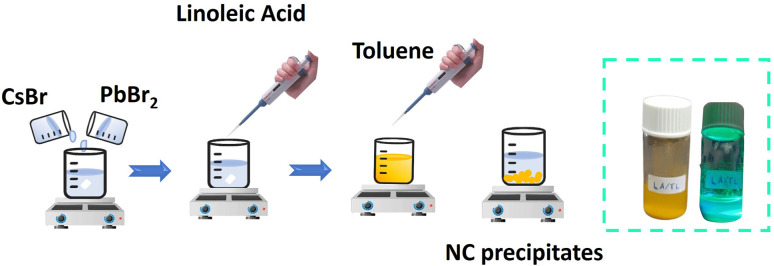
Synthesis process of cesium lead bromide perovskite NCs *via* the rapid LARP method.

Fig. 1 schematically shows the main steps of the LARP method. This method is different from the hot injection method because the reaction takes place at room temperature (∼28 °C). Therefore, the crystal growth is accelerated by the addition of anti-solvent at the end of the synthesis process. In the present study, only the LA ligand was used in the synthesis. OlAm ligand, which is commonly reported in the literature,^37,38^ was not used because OlAm needs a high temperature to be completely removed from nanoparticle surfaces.^39^ High temperature treatment above 100 °C may change the crystal structure of the resulted cesium lead bromide perovskites.^40^ For preparing the precursor solution, CsBr (0.5 mol) and PbBr_2_ (0.5 mol) were separately dissolved in a DMF : DMSO (7 : 3 v/v) mixed solvent. Subsequently, the CsBr solution was added dropwise to the PbBr_2_ solution while stirred until a 1 : 1 v/v was achieved. The solution was then continuously stirred for 30 min. The LA ligand was then added to the precursor solution with the volume ratio of the LA ligand and the precursor solution of 1 : 2. The solution was stirred again for 15 min. Finally, toluene, as the antisolvent was added dropwise to the precursor solution to initiate the formation of the cesium lead bromide NCs.^27,37^ The precipitated powder was dried in a vacuum chamber at 100 °C until the remaining solvent completely evaporated. The powder was then stored and labeled as an unpurified NCs, or in other words, NCs with ligands (LARP-WL).

To obtain cesium lead bromide NCs without ligands on their surface, the cesium lead bromide NC products were purified to remove the ligands by using a mixture solution of ethanol and toluene (1 : 1 v/v). The NCs product was added to the solution and then sonicated at 80 W for 10 min using an ultrasonic processor. A centrifuge was used to collect the cesium lead bromide NCs from the dispersion solution. Finally, these purified NCs were also dried in a vacuum chamber at 100 °C to evaporate the remaining solvent. Herein, this purified NCs sample is referred to as NCs with purification, or without ligands (LARP-WOL). The average crystal sizes of these NCs without and with purification were estimated to be 29.64 nm for LARP-WL and 33.22 nm for LARP-WOL. These crystal sizes were estimated from the FWHMs of the XRD peak by using the Debye–Scherrer equation.^41^

#### 2.2.2 URSOA method

**Fig. 2:**
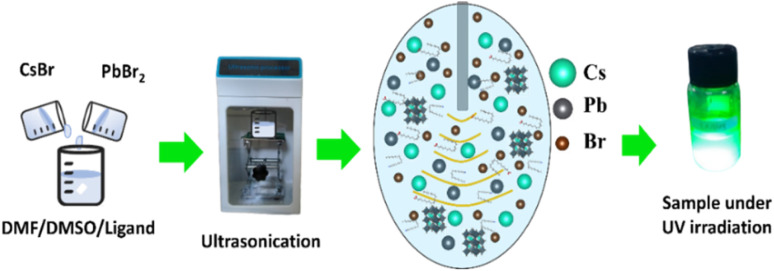
The URSOA synthesis process of cesium lead bromide NCs.

Fig. 2 schematically shows the main steps of the URSOA method. The URSOA method involves synthesis steps similar to those of the LARP method. However, in the URSOA method, instead of the use of anti-solvent, ultrasonication is used to initiate the seed formation of NCs. The synthesis process was initiated by mixing the precursors CsBr and PbBr_2_ in the same molar ratio, followed by the addition of LA/OlAm ligands (LA : OlAm = 1 : 1 v/v) in a precursor-to-ligand ratio of 2 : 1 v/v. Subsequently, the solution mixture was ultrasonicated at 90 W for 10 min using an ultrasonic processor. Similarly to the final synthesis step of the LARP samples above, to obtain the NC products, the precipitate and solvent were separated using a centrifuge and vacuum dryer at 100 °C. The products were referred to NCs with ligands (URSOA-WL). A similar synthesis process was also performed without ligands, where the products referred to as NCs without ligands (URSOA-WOL). The average crystallite sizes were estimated to be 34.64 nm for NCs with ligands (URSOA-WL) and 38.28 nm for NCs without ligands (URSOA-WOL). The names of the synthesized samples and the differences in their synthesis processes are summarized in Table 1.

**Table 1:** Sample names and their differences^a^

Sample name	Synthesis method	LA ligand	OlAm ligand	Anti-solvent	Purification	Ultra-sonication	With ligands
LARP-WL	LARP	✓	✗	Toluene	✗	✗	✓
LARP-WOL	LARP	✓	✗	Toluene	✓	✗	✗
URSOA-WL	URSOA	✓	✓	✗	✗	✓	✓
URSOA-WOL	URSOA	✗	✗	✗	✗	✓	✗

a Note: ✗ indicates that the corresponding process or chemical/ligand was not used, while ✓ indicates that it was used.

### 2.3. Characterization methods

The XRD measurements were performed on dried powder for all samples of cesium lead bromide NCs using the SmartLab X-ray diffractometer (Rigaku, Japan). The wavelength was 1.5406 Å (Cu-Kα radiation). XPS and HAXPES measurements were performed using the Quantes (ULVAC-PHI). Al Kα (*hv*: 1486.6 eV) and Cr Kα (*hv*: 5414.8 eV) sources were used for XPS and HAXPES measurements, respectively. The total energy resolutions were 0.51 and 0.76 eV, respectively. The binding energies were calibrated using the binding energy of the C 1s core-level of the C–H bond of organic molecules (284.8 eV).^42^ To investigate the adsorbed states of the ligands of the NCs, Fourier Transform Infrared (FTIR) measurements (FTIR Alpha II, Bruker) were carried out utilizing the attenuated total reflection mode. The photoluminescence spectra of the cesium lead bromide NCs were measured using an Ocean Optics USB 2000 spectrometer with a 406 nm light source laser. The PL decays were measured using an experimental setup consisting of a pico-second laser (PicoQuant) at 420 nm (with 20 ps pulse width, 50 mW light power, and 10 MHz repetition rate), a photon microdevice detector, and a data acquisition interface (TimeHarp 260 from PicoQuant).^43^

## 3. Results and discussion

### 3.1. Crystal structures, chemical states, and PL characteristics of cesium lead bromide NCs prepared by LARP method

#### 3.1.1 Crystal structures and chemical states

**Fig. 3:**
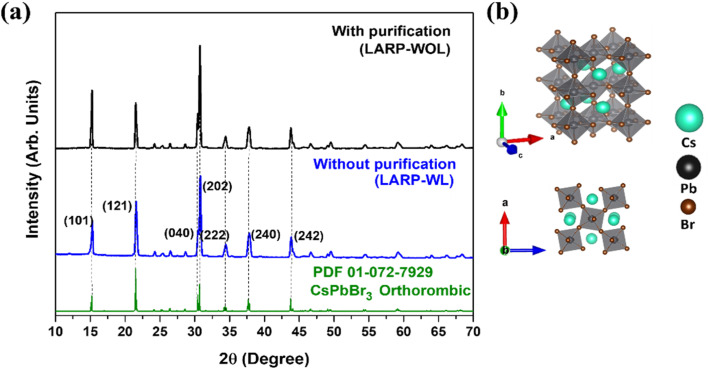
(a) XRD patterns of the NCs with purification (LARP-WOL) and without purification (LARP-WL) and the reference data of the orthorhombic CsPbBr_3_ (PDF-01-072-7929). (b) The top and the side views of crystal structure of orthorhombic CsPbBr_3_.

The XRD measurements were performed to identify the crystal structures of the NCs prepared by the LARP method (LARP WOL and LARP-WL). The XRD patterns of both NCs, as shown in Fig. 3(a), indicate the prominent peaks at 2*θ* = 15.2°, 21.6°, 30.5°, and 30.8°, which could be assigned to the (101), (121), (040), and (202) diffraction planes of orthorhombic CsPbBr_3_ by referring the powder diffraction file PDF-01-072-7929 shown in Fig. 3(a).^44^ The crystal structure of the orthorhombic CsPbBr_3_ is shown in Fig. 3(b). The lattice constants were estimated to be *a* = 8.24 Å, *b* = 11.74 Å, and *c* = 8.2 Å. Because both NCs have the indistinguishable XRD patterns, the orthorhombic crystal structure remains unchanged after the purification process. However, in general, the XRD data respresents the bulk properties of the NCs, which is consequently insensitive to the crystal structure changes in the surface.

**Fig. 4:**
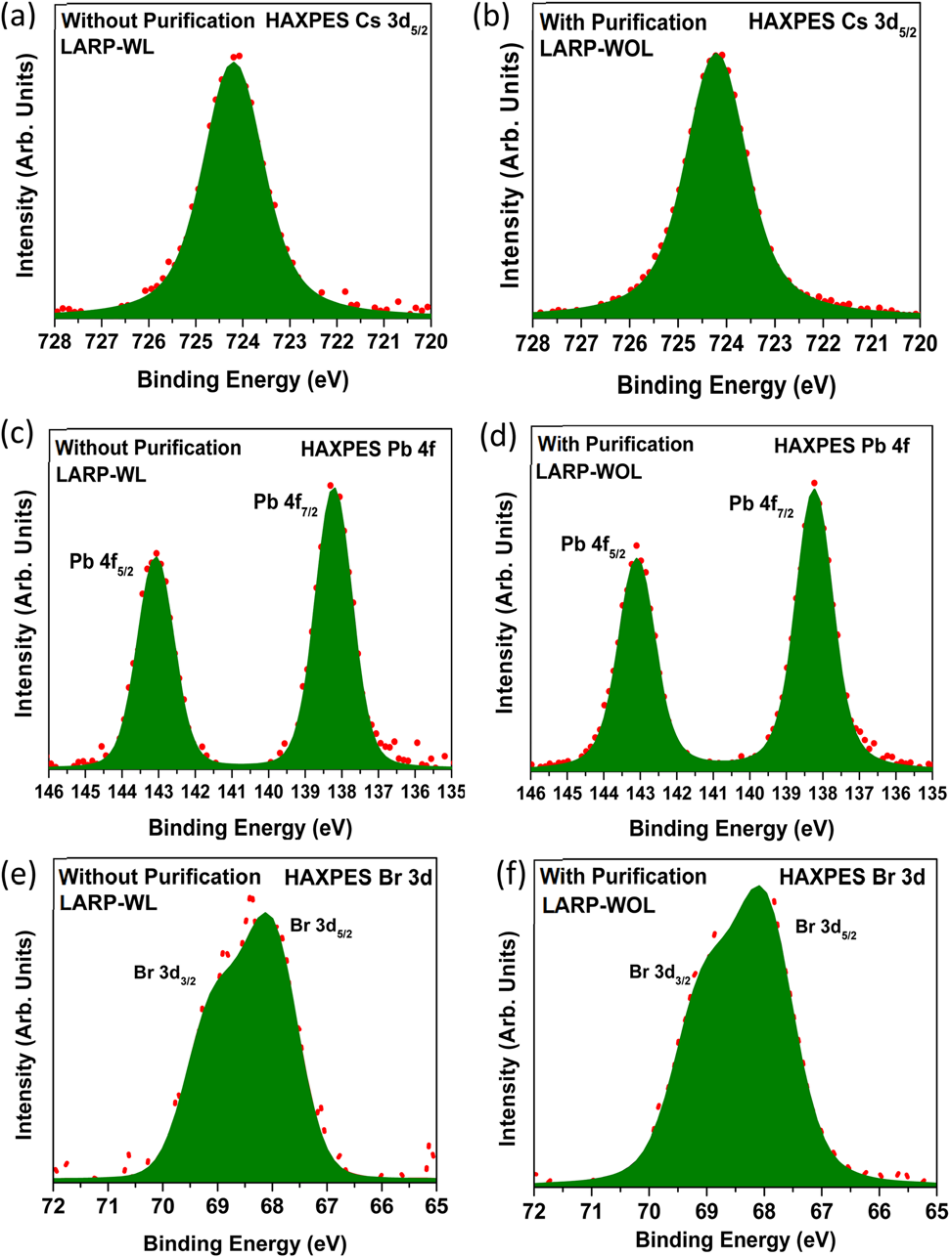
HAXPES spectra of the NCs without purification (LARP-WL) and with purification (LARP-WOL) (a) and (b) for Cs 3d_5/2_; (c) and (d) for Pb 4f; and (e) and (f) for Br 3d, respectively.

Fig. 4(a) and (b) show the Cs 3d_5/2_ core-level HAXPES spectra for the NCs without and with purification (LARP-WL and LARP-WOL), respectively. Both NCs exhibit one peak at 724.2 eV, which is attributed to the Cs atoms in the CsPbBr_3_ NCs.^45,46^Fig. 4(c) and (d) show the Pb 4f core-level HAXPES spectra for the NCs without and with purification (LARP-WL and LARP-WOL), respectively, which show two peaks at Pb 4f_7/2_ (138.2 eV) and Pb 4f_5/2_ (143.1 eV). These peaks are attributed to the Pb atoms in CsPbBr_3_ NCs.^47^Fig. 4(e) and (f) show the Br 3d core-level HAXPES spectra for the NCs without and with purification (LARP-WL and LARP-WOL). Both samples have one component (Br 3d_5/2_ (68.2 eV) and Br 3d_3/2_ (69.2 eV)), which is due to the Br atoms in CsPbBr_3_ NCs.^27,48^ Because HAXPES exhibits bulk chemical information (the information depth of ∼10 nm^49^), the chemical states of the inner side of the NCs might not be affected by the surface ligands and the purification processes.

**Fig. 5:**
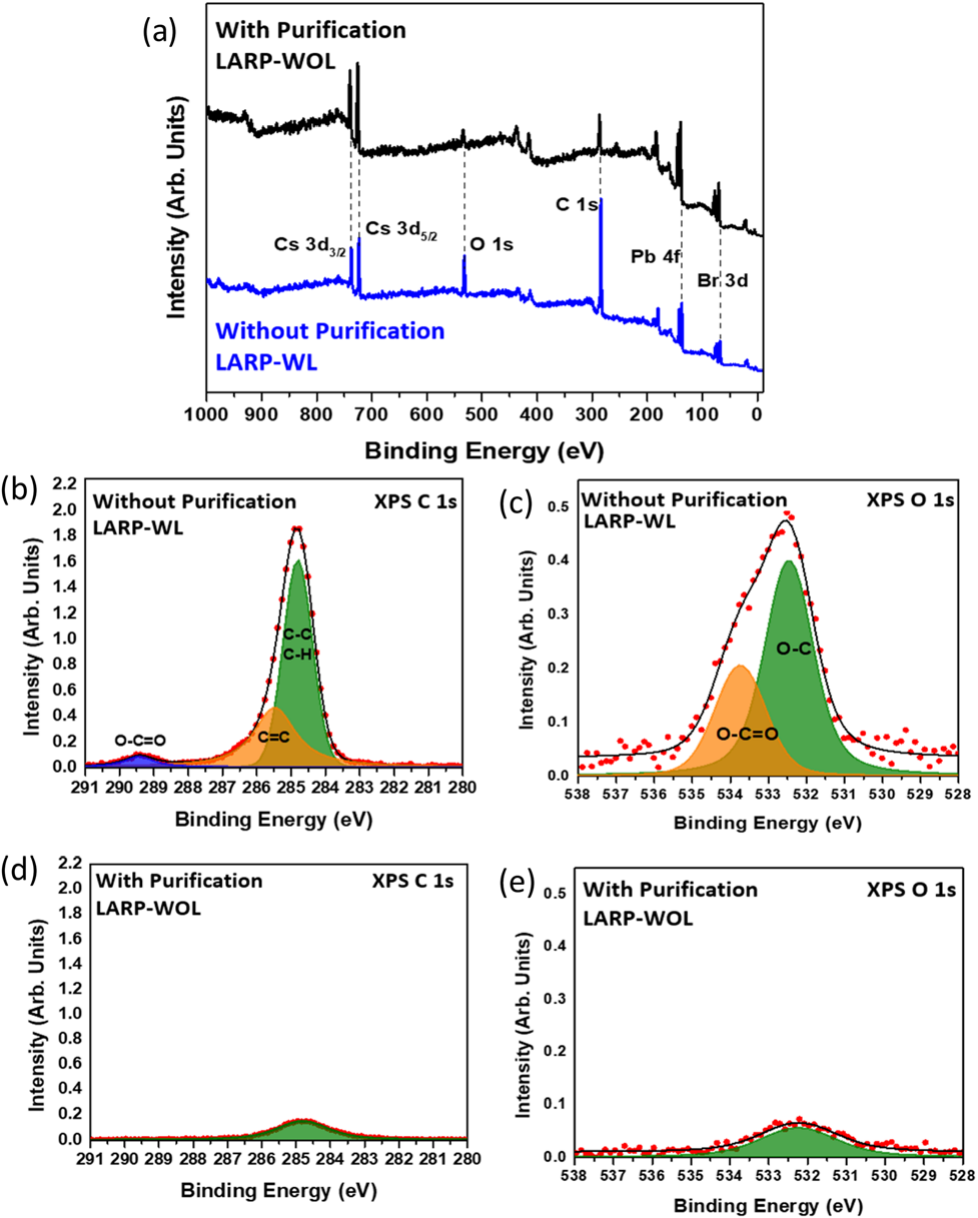
(a) XPS spectra survey for the NCs without purification (LARP-WL) and with purification (LARP-WOL) (b) C 1s and (c) O 1s of the NCs without purification (LARP-WL). (d) C 1s and (e) O 1s of the NCs with purification (LARP-WOL).

XPS measurements were performed to investigate the surface chemical states of the NCs. It should be noted that XPS exhibits relatively surface sensitive characterization method in which the information depth is ∼3 nm.^50^Fig. 5(a) shows the survey XPS spectra of both NCs, showing the presence of Cs, Pb, Br, C, and O atoms in the CsPbBr_3_ NCs. The individual survey spectra of these NCs are shown in Fig. S1 in the SI. These NCs show significant differences for the peak intensities of the C 1s and O 1s core-levels, where the NCs without purification (LARP-WL) show higher peak intensities of C 1s and O 1s than the NCs with purification (LARP-WOL). Fig. 5(b) shows the C 1s spectrum of the NCs without purification (LARP-WL), which can be deconvoluted into three components. The peak at 284.8 eV is attributed to C–C and C–H bonds of the LA molecules, whereas the peaks at 285.3 eV and 289.3 eV are associated with C=C and O–C=O bonds of the LA molecules, respectively.^51^ For the O 1s spectrum (Fig. 5(c)), there are two components observed at 532.4 eV and 533.7 eV, which are attributed to O–C and O–C=O bonds of the LA molecules, respectively.^51,52^Fig. 5(d) and (e) show the C 1s and O 1s core-level XPS spectra for the NCs with purification (LARP-WOL), showing a drastic decrease in the areal peak intensities comparing to the case of the NCs without purification (LARP-WL). Therefore, the ligands at the CsPbBr_3_ NC surface are thoroughly removed by the purification process. The peaks at 284.4 eV and 532.3 eV for respective C 1s and O 1s core-levels may be attributed to the surface contaminations formed during sample preparation process,^53^ which are usually observed in XPS spectra.^54^

**Fig. 6:**
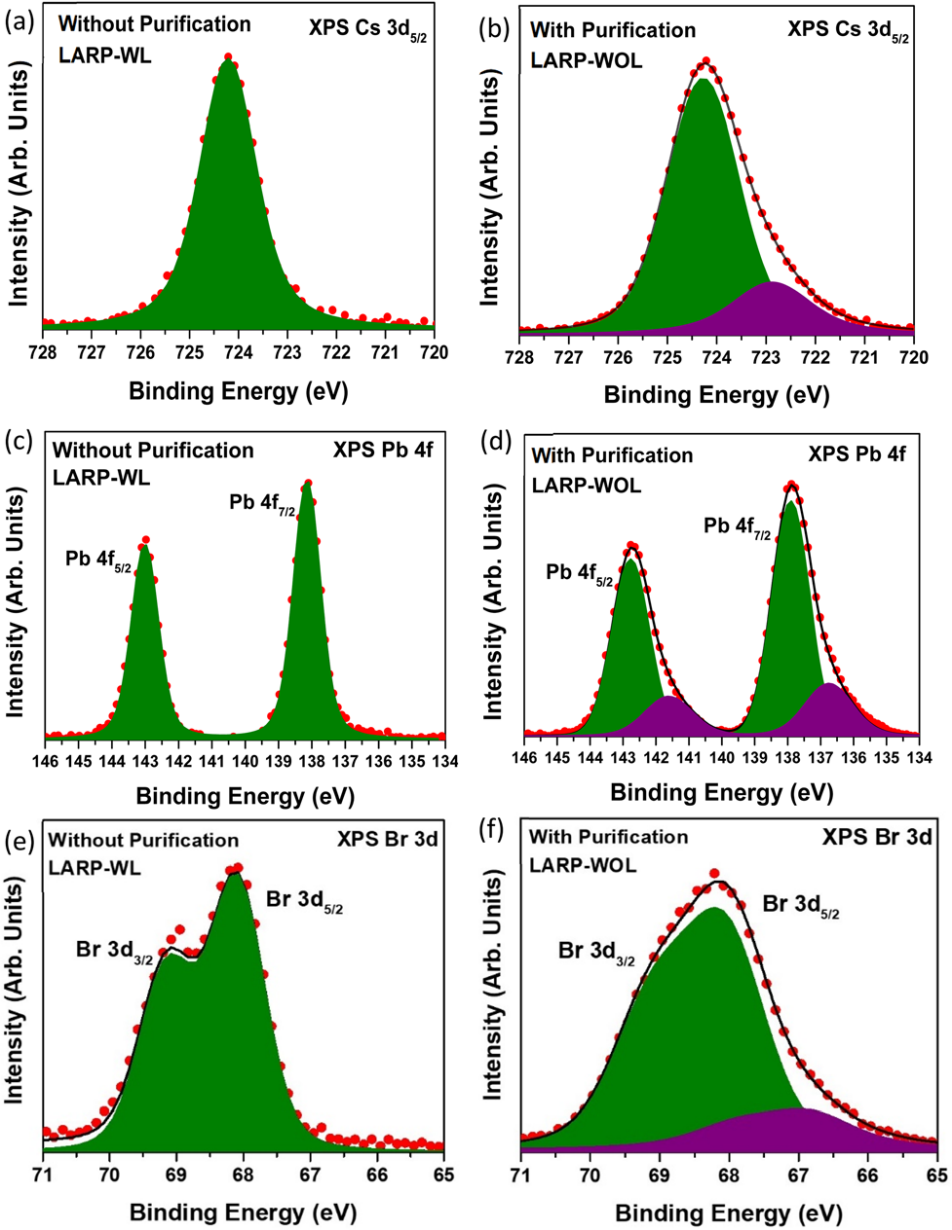
XPS spectra of the NCs without purification (LARP-WL) and with purification (LARP-WOL); (a) and (b) for Cs 3d_5/2_; (c) and (d) for Pb 4f; and (e) and (f) for Br 3d, respectively.

Fig. 6(a) shows the Cs 3d_5/2_ core-level XPS spectrum of the NCs without purification (LARP-WL). There is one peak at 724.2 eV, which is attributed to the Cs atoms in CsPbBr_3_ NCs.^45,46^Fig. 6(b) shows the Cs 3d_5/2_ core-level XPS spectrum of the NCs with purification (LARP-WOL). There are two components at 724.2 and 722.9 eV. The higher binding energy component at 724.2 eV (green color shown in Fig. 6(b)) could be attributed to the Cs atom in CsPbBr_3_ NCs,^45,46^ whereas the lower-binding-energy component at 722.9 eV (purple color) might be due to Cs^+^ accumulation at the surface.^55^Fig. 6(c) and (d) show the Pb 4f core-level spectra of the NCs without and with purification. The NC without purification (LARP-WL) (Fig. 6(c)) has one component (Pb 4f_7/2_ (138.2 eV) and Pb 4f_5/2_ (143.1 eV)), which is attributed to the Pb atoms in CsPbBr_3_ NCs.^47^ In contrast, the NC with purification (LARP-WOL) (Fig. 6(d)) has two components. The higher binding energy component (Pb 4f_7/2_ (138 eV) and Pb 4f_5/2_ (142.9 eV)) is attributed to the Pb atom in CsPbBr_3_ NCs, whereas the lower binding energy state (Pb 4f_7/2_ (136.8 eV) and Pb 4f_5/2_ (141.7 eV)), might be due to Pb atom with oxidation state of zero (Pb^0^) which might not bond with the Br atoms and could be present at the NCs surface.^55–57^

The Br 3d core-level XPS spectra for the NCs without and with purification (respective LARP-WL and LARP-WOL) are shown in Fig. 6(e) and (f). For the NCs without purification (LARP-WL) (Fig. 6(e)), the Br 3d core-level spectrum has one component (green color). The green component at 68.2 eV (Br 3d_5/2_) and 69.2 eV (Br 3d_3/2_) could be originated from the Br atoms in CsPbBr_3_ NCs.^27,48^ In contrast, for the NCs with purification (LARP-WOL) (Fig. 6(f)), the Br 3d XPS spectrum shows two components. The component at 68.0 eV (Br 3d_5/2_) and 69.0 eV (Br 3d_3/2_) (green color) could be originated from the Br atom in the CsPbBr_3_ NCs.^27,48^ The lowest binding energy component at 66.8 eV (Br 3d_5/2_) and 67.8 eV (Br 3d_3/2_) (purple color) might be due to unbonded Br atoms present at the NC surface.^58^ All core-level spectra were fitted using a Voigt profile function, and the detailed fitting parameters are provided in the SI (Tables S1 and S2).

**Fig. 7:**
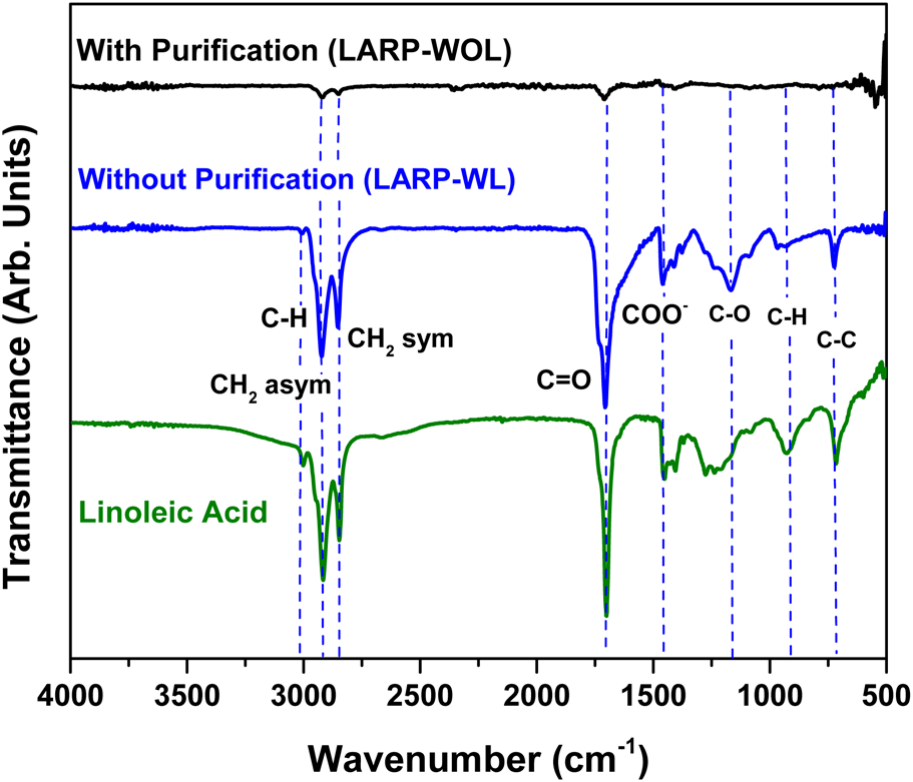
FTIR spectra of the NCs without purification (LARP-WL) and with purification (LARP-WOL). The FTIR spectrum of the LA ligand is also shown as a reference in the vibration component assignments.

In order to investigate the chemical species of the ligands before and after purification, the FTIR measurements were performed for those NCs prepared by LARP methods. Fig. 7 shows the FTIR spectra of the NCs without purification (LARP-WL) and with purification. (LARP-WOL). For the NCs without purification (LARP-WL), a strong vibration peak at 1712.9 cm^−1^ is attributed to the C=O (carbonyl) stretching vibration mode of the LA molecules whereas the peak at 2850–2924 cm^−1^ is due to symmetric and asymmetric vibrations of CH_2_ groups.^38,59,60^ The peak at 3012 cm^−1^ is originated from C–H stretching in the C=C–H species.^61^ For the NCs with purification (LARP-WOL), although the intensities of those vibration bands are drastically decreased, several vibration bands such as C=O (at 1710.5 cm^−1^) and C–H vibrations (at 2848.6 cm^−1^ and 2921.6 cm^−1^) are still observed, indicating that an extremely small residue of the LA molecules remains after the purification. Further the details in the vibration band assignments of these FTIR spectra are shown in the Table S3. Note that these FTIR results are consistent with the C 1s XPS results described above, where significantly weak components of C 1s and O 1s remain after purification process (LARP-WOL) as shown in Fig. 5(a).

**Fig. 8:**
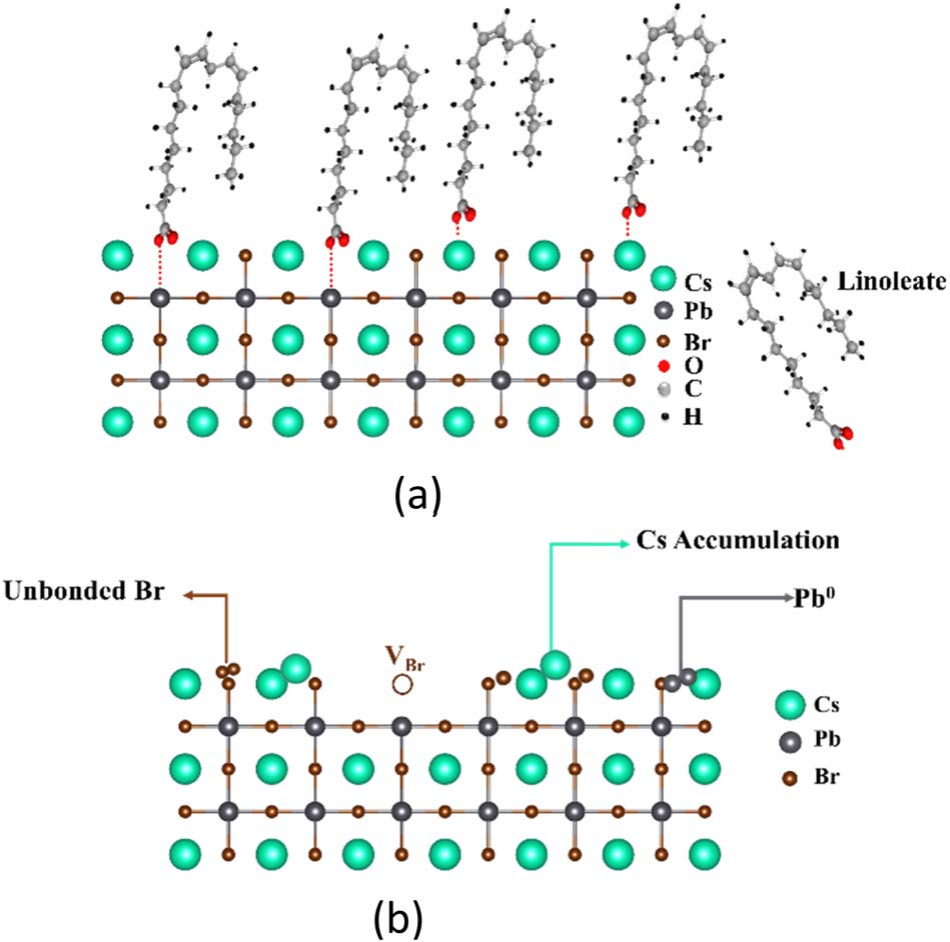
Possible structures of the NCs near the surface (a) before and (b) after purification.

From XRD, HAXPES, XPS, and FTIR results, the structural models before and after purification are proposed in Fig. 8(a) and (b). Before purification (Fig. 8(a)), the LA ligands are attached to the surface of the NCs. The carboxylate group of the ligands, which are negatively charged (COO^−^),^62^ might bond to the positively charged Pb^2+^ and Cs^+^ at the NC surface. As a result, the ligands could passivate the surface atoms, thus preventing the formation of defect species at the NC surface. After the purification process (Fig. 8(b)), the ligands are removed leading to termination of the crystal periodicity at the NC surface, thus forming the accumulation of Cs cations, Pb^0^, and unbonded Br. In addition, the Br anions might also be detached from the NC surface after purification, resulting in the formation of Br vacancies. Our results suggest that the ligands might effectively prevent the formation of defect species at the NCs surface.

#### 3.1.2 PL characteristics

**Fig. 9:**
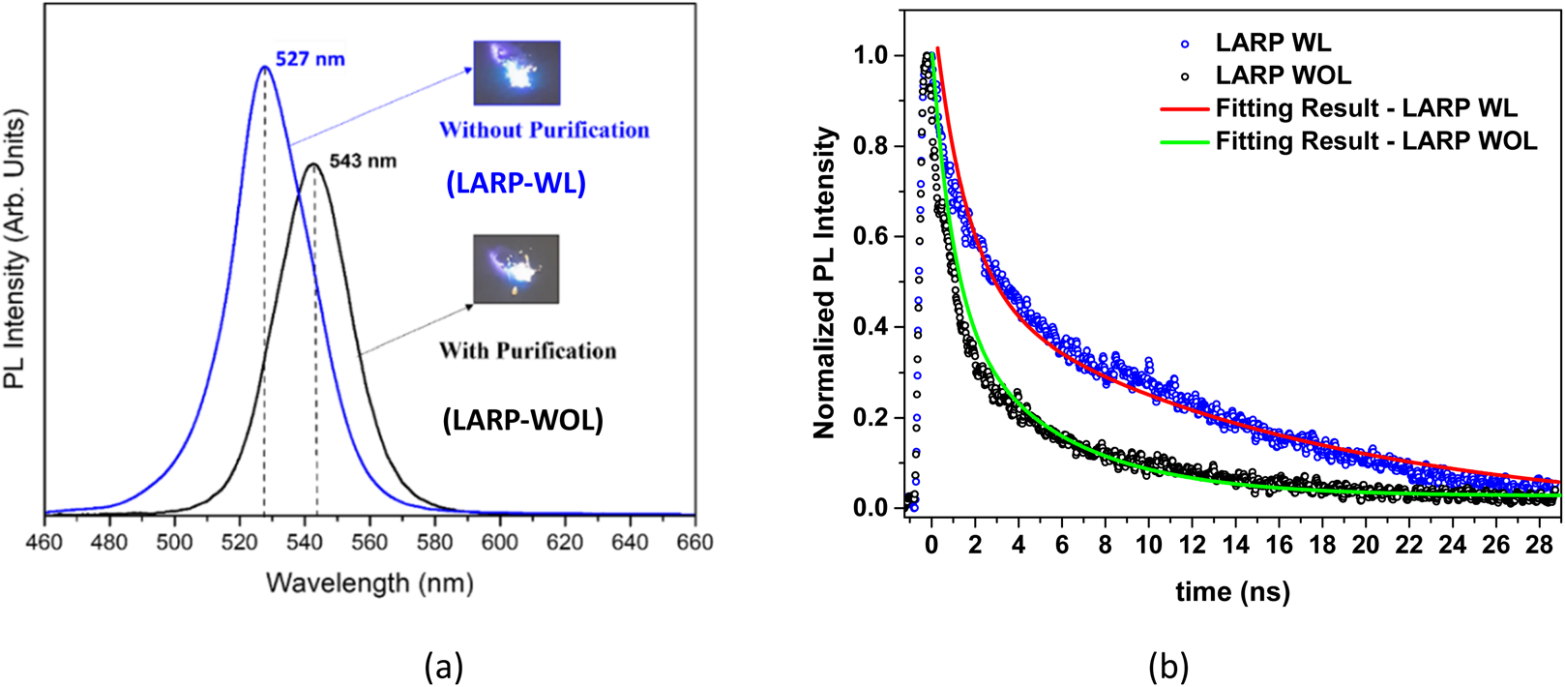
(a) PL spectra of the NCs without purification (LARP-WL) and with purification (LARP-WOL). (b) The normalized intensity of PL as a function of time measured for the LARP-WL and LARP-WOL NCs. The fitting results are shown as solid lines.

Fig. 9(a) shows the PL spectra of the NCs without purification (LARP-WL) and with purification (LARP-WOL). The NCs without purification (LARP-WL) exhibit a shorter wavelength peak and higher PL intensity compared to the NCs with purification (LARP-WOL). Weak PL intensity observed at the NCs without purification (LARP-WL) might be attributed to the surface defect species,^63,64^ such as the accumulation of Cs^+^, Pb^0^, unbonded Br atoms, and Br vacancies at the NC surface. These defect species might form trap states in the bandgap and act as non-radiative recombination centers, decreasing the PL intensity.^65^ For the observed red-shift peak, Liu *et al.* have conducted computational studies on the electronic structure of CsPbBr_3_ NCs and have shown that CsPbBr_3_ NCs with Br vacancies have a smaller band gap in comparison to the defect free CsPbBr_3_ NCs.^66^ Thus, the observed red-shift for the NCs with purification (LARP-WOL) may be thus caused by the Br vacancies formed at the NC surface.^67^

Fig. 9(b) shows the PL intensity as a function of time (decay) for NCs with and without purification, indicating that the PL decay of the NCs with purification (LARP-WOL) exhibits more rapid decay compared to the case of the NCs without purification (LARP-WL). According to the previous studies, the PL intensity as a function of time (decay) can be fitted with a bi-exponential function consisting of the fast and the slow decay components, which is given by^68,69^1
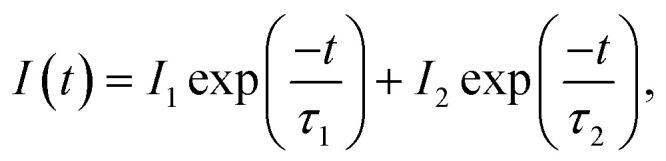
where *I*(*t*) represents the PL intensity at the time *t*, *I*_1_ and *I*_2_ denote the initial intensities of the fast and the slow decay components, and *τ*_1_ and*τ*_2_ denote the decay time constants of the fast and the slow decays, respectively. The average decay time (*τ*_avg_) can be calculated using the following equation:^69^2
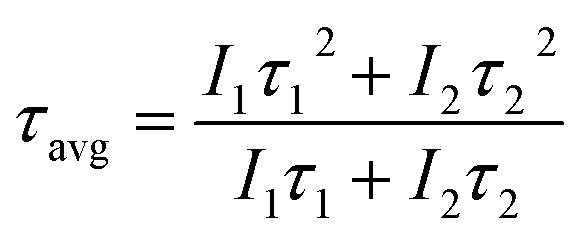


The decay parameters obtained from fitting results are shown in Table 2. The fast decay component could be ascribed to the radiative recombination of free excitons, whereas the slow decay could be associated with the radiative recombination of trapped excitons.^70,71^ Longer decay times for the NCs without purification (LARP-WL) maybe an indicative of predominant radiative recombinations of excitons. In contrast, faster decays in the NCs with purification (LARP-WOL) may indicate pre-dominant non-radiative recombinations of excitons *via* defect states.

**Table 2:** The fitting results obtained from curve fittings of PL decays in Fig. 9 using a bi-exponential decay function. *I*_1_ and *I*_2_ are the normalized initial intensities

Sample	*I*_1_	*τ*_1_ (ns)	*I*_2_	*τ*_2_ (ns)	*τ*_avg_ (ns)
NCs without purification (LARP-WL)	0.58	1.77	0.50	14.12	12.55
NCs with purification (LARP-WOL)	0.56	0.98	0.43	5.02	4.20

According to the previous report, the fast decay originates from the radiative recombination of free exciton occuring at the inner side of NCs.^70^ On the other hand, the slow decay originates from the radiative recombination of trapped exciton occuring mainly at the NC surface.^71^ In the case of the NCs with purification (LARP-WOL), the surface defect species, such as accumulation of Cs, Pb^0^, the unbonded Br atoms, and the Br vacancies, could act as non-radiative centers for excitons, resulting in a shorter lifetime of radiative recombination and predominance of non-radiative recombination. A similar trend was also observed in the average decay times, where NCs without purification (LARP-WL) is longer than NCs with purification (LARP-WOL), indicating that ligands effectively suppress non-radiative recombination pathways.

### 3.2. Crystal structures, chemical states and PL characteristics of cesium lead bromide NCs prepared by URSOA

#### 3.2.1 Crystal structures and chemical states in the NCs

**Fig. 10:**
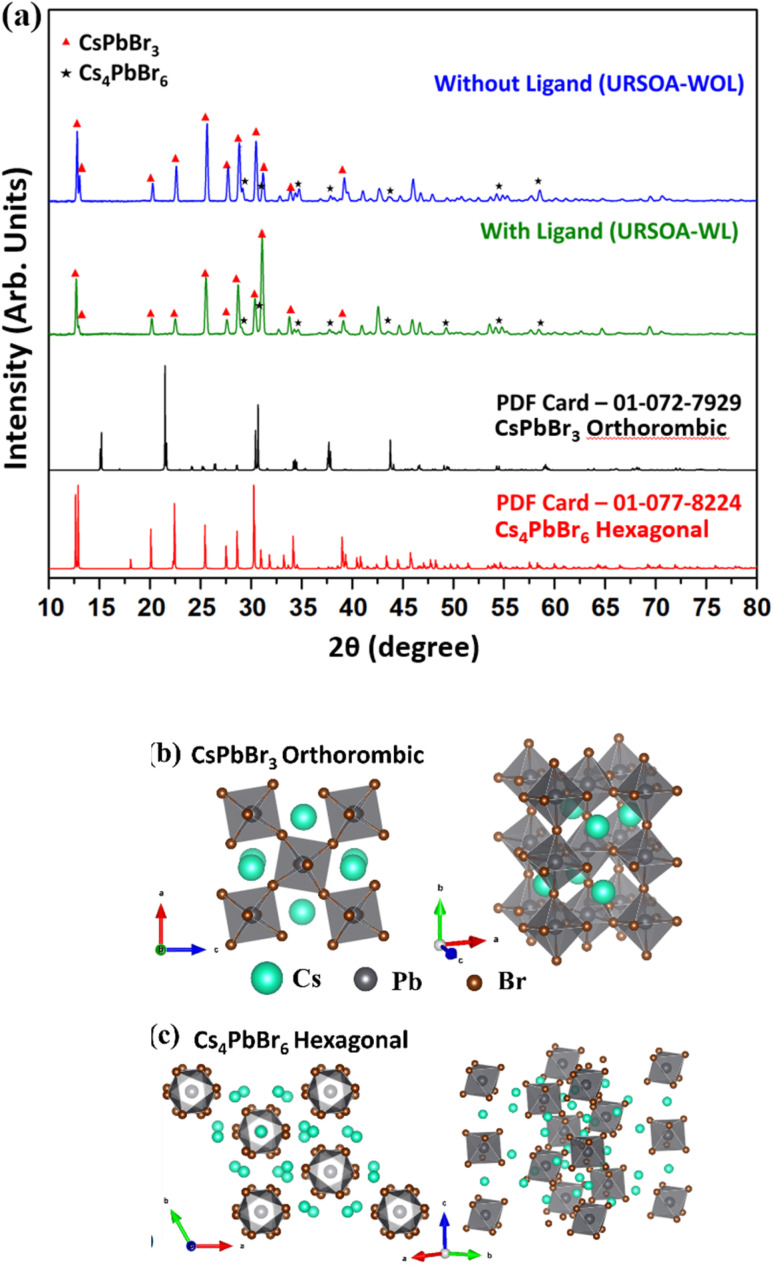
XRD patterns of the NCs without (blue line) and with (green line) ligands. The (★) symbol indicates the diffraction peak from CsPbBr_3_ phase, whereas the (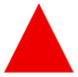) symbol indicates the diffraction peak from Cs_4_PbBr_6_ phase. Reference data for orthorhombic CsPbBr_3_ (PDF-01-072-7929) (black line) and hexagonal Cs_4_PbBr_6_ (PDF-01-077-8224) (red line) are also shown for comparison. (b) The orthorhombic crystal structure of CsPbBr_3_ (*Pnma* space group) and (c) the hexagonal crystal structure of Cs_4_PbBr_6_ (*R*3̄*c* space group).

The XRD patterns of the NCs without and with ligands prepared by the URSOA method (URSOA-WOL and URSOA-WL) are shown in Fig. 10(a). These NCs show not only the indistinguishable XRD patterns of the NCs prepared by the LARP method but also exhibit additional XRD peaks observed only by the URSOA method. By comparison with the reference XRD data PDF-01-077-8224, the diffraction peaks at 12.7°, 12.9°, 20.1°, 22.5°, 25.5°, 27.7°, 28.6°, 30.3°, 30.9°, 34.2° and 38.9° could be assigned to the crystal planes (012), (110), (113), (300), (024), (131), (214), (223), (006), (134), and (324) of Cs_4_PbBr_6_ with hexagonal crystal structure.^18,69^ The lattice constant was estimated to be *a* = *b* = 13.72 Å and *c* = 17.32 Å, with *α* = *β* = 90° and *γ* = 120°.^72,73^ In addition, in comparison with PDF-01-072-7929, the peaks at 29°, 30.7°, 34.6°, and 43.7° can be assigned to the (122), (202), (103), and (242) planes of the orthorhombic CsPbBr_3_ crystal structure.^27,69^ The lattice constants were estimated to be *a* = 8.24 Å, *b* = 11.74 Å, and *c* = 8.2 Å. These XRD patterns indicate the formation of both CsPbBr_3_ and Cs_4_PbBr_6_ crystal phases in the NCs prepared by URSOA method.

It should be noted that CsPbBr_3_ exhibits a three-dimensional (3D) perovskite structure with orthorhombic structure (*Pnma* space group) at room temperature. This phase composes of a continuous framework of corner-sharing [PbBr_6_]^4−^ octahedra, where Cs^+^ ions occupy the interstitial A-site positions within the perovskite framework,^74^ as shown in Fig. 10(b). In contrast, Cs_4_PbBr_6_ has a hexagonal crystal structure (*R*3̄*c* space group),^18,75^ like an array or cluster of isolated [PbBr_6_]^4−^ octahedra groups connected with Cs^+^ ions, which act as a spacer to maintain the separation between octahedra, as shown in Fig. 10(c).^18^ Therefore, regardless of the presence of the ligands, two distinct phases of the perovskite crystals are formed, namely orthorhombic CsPbBr_3_ and hexagonal Cs_4_PbBr_6_ crystal structures. By performing quantitative phase-analysis using the Rietveld method, the XRD patterns can be analyzed to determine the proportion of each phase.^76^ The phase molar ratios of Cs_4_PbBr_6_ to CsPbBr_3_ for the NCs with and without ligands (respective URSOA-WL and URSOA-WOL) were estimated to be 10.1 : 1 and 11.5 : 1, respectively, which are relatively in good agreement with the other studies.^69,77,78^

**Fig. 11:**
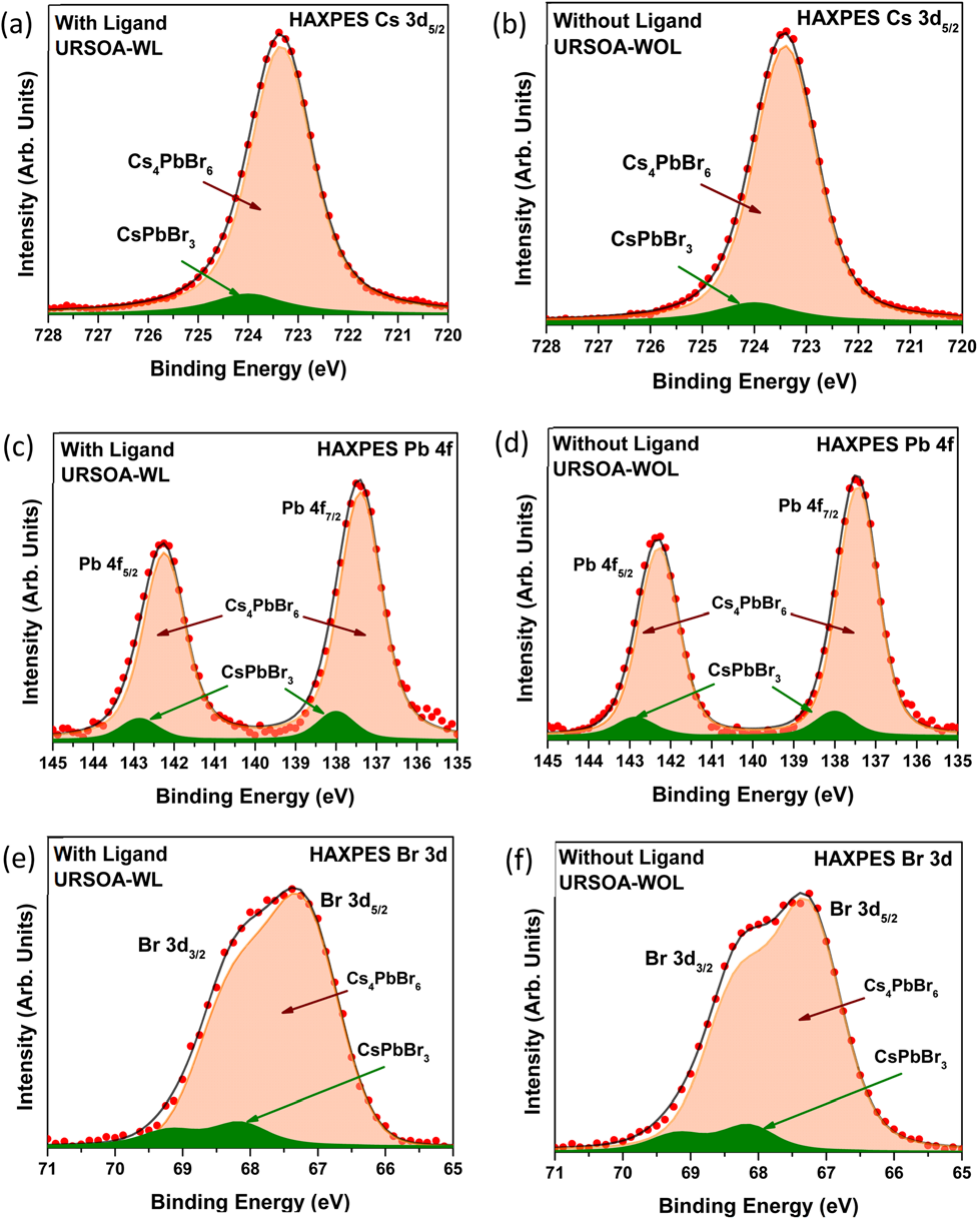
HAXPES spectra for the NCs with ligands (URSOA-WL) and without ligands (URSOA-WOL): (a) and (b) for Cs 3d;_5/2_ (c) and (d) for Pb 4f; and (e) and (f) for Br 3d, respectively.

Fig. 11(a) and (b) show the Cs 3d_5/2_ core-level HAXPES spectra for the NCs with and without ligands (URSOA-WL and URSOA-WOL). These HAXPES spectra were fitted by referring to the phase molar ratios of Cs_4_PbBr_6_ : CsPbBr_3_, based on the XRD analysis results above, at 10.1 : 1 for URSOA-WL and 11.5 : 1 for URSOA-WOL. The component at 724 eV (green color) can be assigned to the Cs atoms in the CsPbBr_3_ NCs, whereas the lower binding energy component at 723.3 eV (cream color) can be assigned to the Cs atoms in the Cs_4_PbBr_6_ NCs.^45,46,79^Fig. 11(c) and (d) show the Pb 4f core-level HAXPES spectra for the NCs with and without ligands (URSOA-WL and URSOA-WOL), which also indicate that the Pb atoms exhibit two different chemical states. The lower binding energy component at 137.4 eV (Pb 4f_7/2_) and 142.3 eV (Pb 4f_5/2_) is attributed to the Pb atoms in the Cs_4_PbBr_6_ NCs,^80^ whereas the higher binding energy component at 138.0 eV (Pb 4f_7/2_) and 142.8 eV (Pb 4f_5/2_) could be due to the Pb atom in the CsPbBr_3_ NCs.^47^Fig. 11(e) and (f) show the Br 3d core-level HAXPES spectra. The component at 67.2 eV (Br 3d_5/2_) and 68.2 eV (Br 3d_3/2_) could be attributed to the Br atoms of the Cs_4_PbBr_6_ NCs,^28,81^ whereas the higher binding energy component (green color) peaked at 68.2 eV (Br 3d_5/2_) and 69.2 eV (Br 3d_3/2_) could be due to the Br atoms of the CsPbBr_3_ NCs.^28^ Therefore, the chemical states of the inner side of the NCs are not affected by the presence and absence of the ligands.

**Fig. 12:**
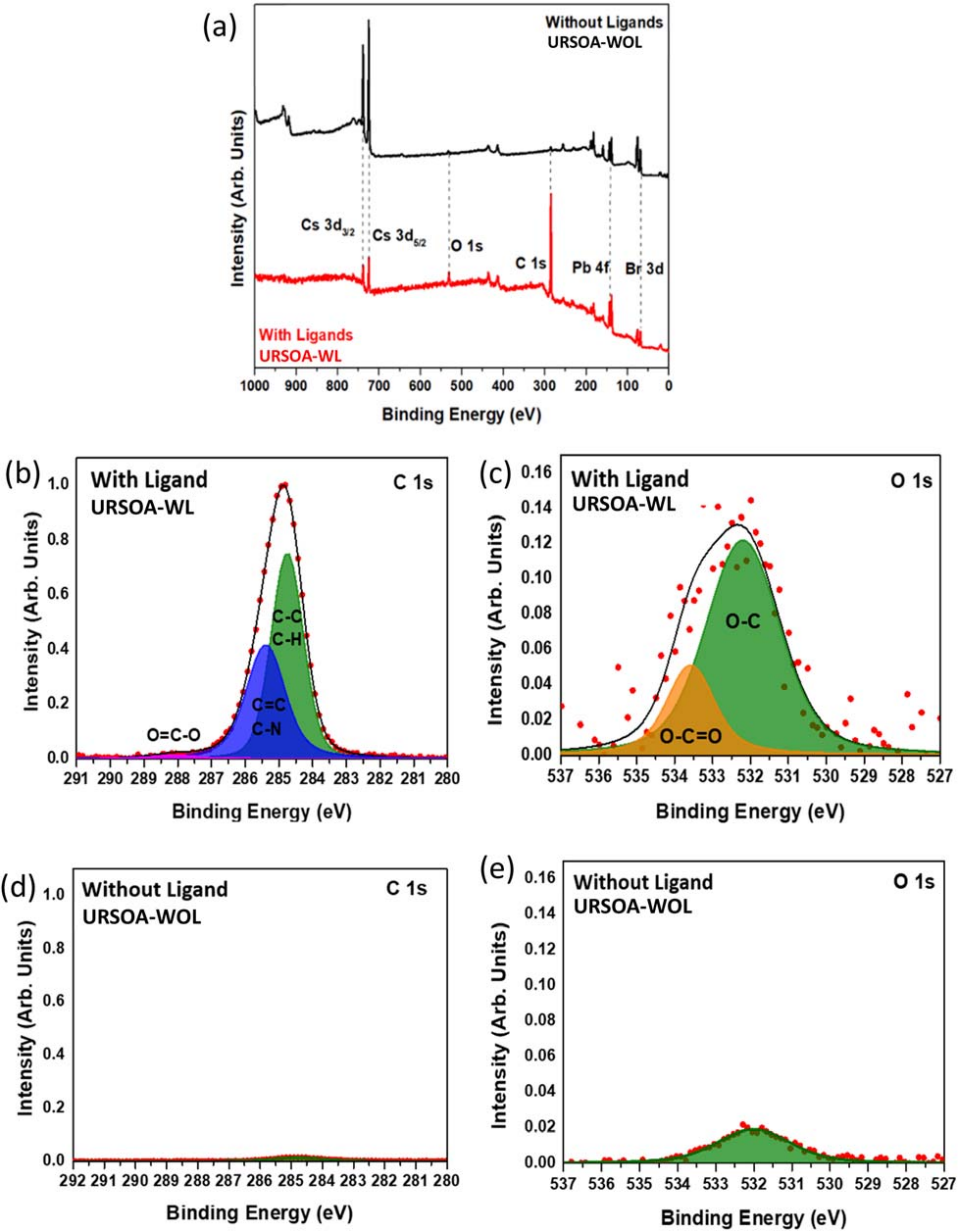
(a) XPS survey spectra for the NCs with ligands (URSOA-WL)and without ligands (URSOA-WOL). XPS spectra for (b) C 1s and (c) O 1s of NCs with ligands (URSOA-WL). XPS spectra for (d) C 1s and (e) O 1s of NCs without ligands (URSOA-WOL).

Fig. 12(a) shows the survey XPS spectrum of the NCs with and without ligands (URSOA-WL and URSOA-WOL), showing a substantial difference in the peak intensities of C 1s and O 1s. The NCs with ligands (URSOA-WL) have much higher peak intensities of C 1s and O 1s than the NCs without ligands (URSOA-WOL). The individual survey spectra of both NCs are shown in Fig. S2 in the SI. Fig. 12(b) shows the C 1s spectrum of the NCs with ligands (URSOA-WL), which can be deconvoluted into three components. The peak at 284.8 eV is attributed to the C atoms of C–C and C–H bonds of the LA and the OlAm molecules, whereas the peaks at 285.4 eV and 288.1 eV are due to the C atoms of C=C/C–N and O–C=O bonds of the LA and the OlAm molecules, respectively.^51^ For the O 1s XPS spectrum of the NCs with ligands (URSOA-WL) (Fig. 12(c)), there are two components at 532.2 eV and 533.6 eV, which might be attributed to O–C and O–C=O bonds of the LA and the OlAm molecules, respectively.^51,52^ The weak peak intensities of the C 1s and O 1s core-levels of the NCs without ligands (URSOA-WOL), as shown in Fig. 12(d) and (e), might be originated from the contaminants formed by sample preparation process.^53^

**Fig. 13:**
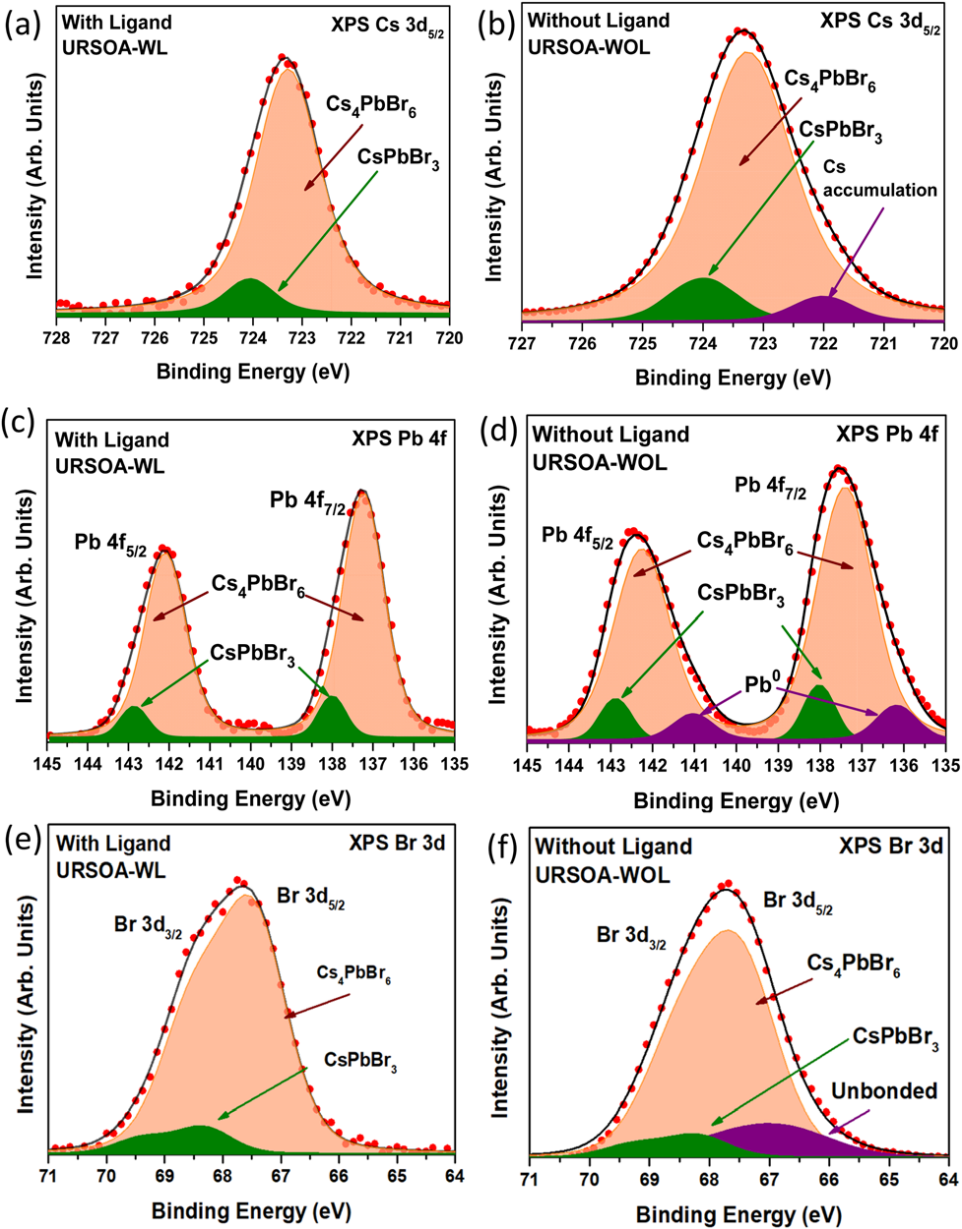
XPS spectra of the NCs with ligands (URSOA-WL) and without ligands (URSOA-WOL); (a) and (b) Cs 3d_5/2_; (c) and (d) Pb 4f; and (e) and (f) Br 3d, respectively.

Fig. 13(a) shows the Cs 3d_5/2_ core-level XPS spectrum for the NCs with the ligands (URSOA-WL). The higher binding energy component at 724 eV is attributed to the Cs atom in the CsPbBr_3_ NCs (green color),^46^ whereas the lower binding energy component at 723.3 eV is due to the Cs atom in Cs_4_PbBr_6_ NCs (cream color).^28^ In contrast, for the NCs without ligands (URSOA-WOL), there are three components as shown in Fig. 13(b). The highest and the second highest binding energy components at 724 eV and 723.3 eV are attributed to the Cs atom in the CsPbBr_3_ and the Cs_4_PbBr_6_ NCs, respectively.^28,46^ The lowest binding energy component at 722 eV (purple color) might be attributed to the accumulation of Cs^+^ at the surface of the NCs.^55^

Fig. 13(c) and (d) show the Pb 4f XPS spectra for the NCs with and without ligands (URSOA-WL and URSOA-WOL). For the NCs with the ligands (URSOA-WL), the highest and the second highest binding energy components at 138.0 eV and 137.3 eV could be attributed to the Pb atoms in the CsPbBr_3_ and Cs_4_PbBr_6_ NCs, respectively.^80^ In contrast, for the NCs without the ligands (URSOA-WOL), the two highest binding energy components can be attributed to the Pb atoms in the CsPbBr_3_ and the Cs_4_PbBr_6_ NCs, respectively. The lowest binding energy component observed at 136.2 eV (purple color) might be attributed to Pb^0^.^47,57^Fig. 13(e) shows the Br 3d spectrum of the NCs with ligands (URSOA-WL). The green color components are attributed to the Br atoms in CsPbBr_3_ NCs,^27,48^ whereas the cream color components might be due to the Br atoms in Cs_4_PbBr_6_ NCs.^28^ On the other hand, for Br 3d spectrum of the NCs without ligands (URSOA-WOL), there is one additional component (purple color) observed at 66.7 eV (Br 3d_5/2_) (Fig. 13(f)), which may be attributed to the unbonded Br atoms present at the NCs surface.^58^ Furthermore, similar to the XPS analysis of LARP NCs, the core-level spectra of URSOA NCs were fitted using the Voigt function and the corresponding parameters are summerized in Tables S4 and S5.

**Fig. 14:**
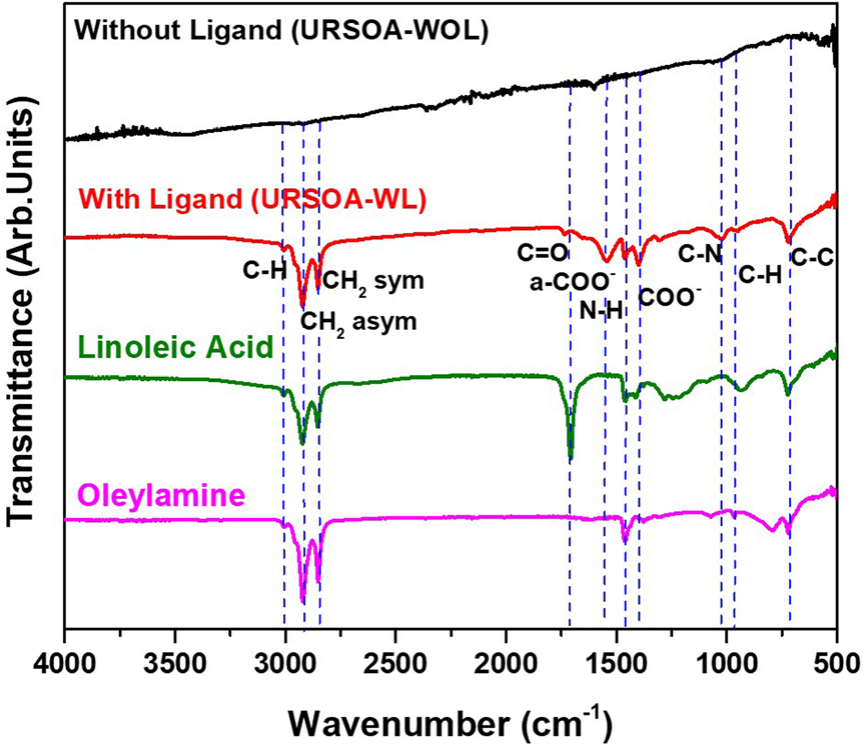
FTIR spectra of the NCs without ligands (URSOA-WOL) and with ligands (URSOA-WL). The FTIR spectra of LA and OlAm ligands are also shown as the references.

Fig. 14 shows the FTIR spectra of the NCs with and without ligands (URSOA-WL and URSOA-WOL). The NCs with ligands (URSOA-WL) show similar vibrational peaks to the LA (green color) and the OlAm (magenta color) molecules, indicating that the LA and the OlAm molecules could bond to the NC surface. The bands at 2919 and 2849 cm^−1^ correspond to asymmetric and symmetric stretching vibration mode of the –CH_2_– groups^38,59,60^ in long-chain alkyl groups of both LA and OlAm molecules. A weak shoulder around 3006 cm^−1^ corresponds to C–H stretching mode.^59^ The vibration peak at 1731 cm^−1^ is attributed to the C=O (carbonyl) stretching vibration mode of the LA molecules. The absorption at 1538 cm^−1^ is attributed to the asymmetric stretching vibration mode of COO^−^ of the carboxylate group, whereas the band around ∼1400 cm^−1^ corresponds to symmetric stretch mode of COO^−^ of the carboxylate group, indicating the deprotonated carboxylic acid of the LA molecules. Additionally, a band around 1500 cm^−1^ is assigned to N–H bending mode,^38,82^ which is due to amine species of the OlAm molecule.^60^ The peak at 1019 cm^−1^ is assigned to the C–N stretching vibration modes of the OlAm molecule.^83^ In contrast, for the NCs without ligand (URSOA-WOL), the vibrational peaks corresponding to the LA and the OlAm molecules are hardly ever observed, indicating the LA and the OlAm molecules could not bond to the NC surface. The detail vibrational band assignments for the FTIR spectra of these samples are provided in the Supplementary Information (Table S6).

**Fig. 15:**
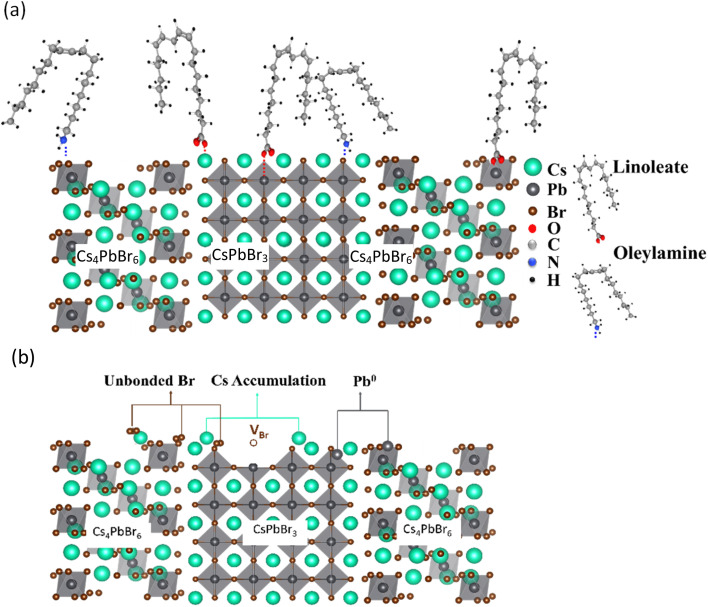
Structural models of the NCs surface (a) with ligands (URSOA-WL) and (b) without ligands (URSOA-WOL).

Fig. 15(a) shows the proposed surface structure of the NCs with ligands obtained from XRD, HAXPES, XPS, and FTIR results. In the case of URSOA-WL, the LA and the OlAm molecules could interact with the NC surface. The carboxylate groups of the LA molecules may form a bonding with the Cs and the Pb cations at the outermost crystal sites, or they may also attach at the Br vacancies on the NC surface.^62^ In the case of the OlAm molecules, the amine group (–NH_2_) might be positively charged due to the formation of –NH_3_^+^,^84,85^ which might bond to the Br anions at the NC surface. The amine group enables the OlAm ligand to bond specifically to Br anion at the outermost NCs surface. Thus, these ligands are able to passivate the surface defects and inhibit the presence of uncoordinated atoms. On the other hand, the NCs without ligand (URSOA-WOL) exhibit the formation of surface defect species such as the accumulation of Cs^+^, Pb^0^, unbonded Br atoms, and Br vacancies at the NC surface (Fig. 15(b)). Therefore, the ligands effectively prevent the formation of defects at the surface of the NCs.

#### 3.2.2 PL characteristics

**Fig. 16:**
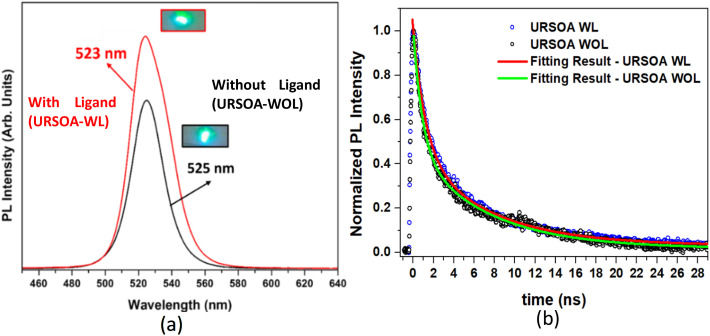
(a) PL spectra of the NCs prepared by URSOA method: the NCs with (red line) and without ligands (black line). (b) The normalized intensity of PL as a function of time measured for the URSOA-WL and URSOA-WOL NCs. The fitting results are also shown as solid lines.

Fig. 16(a) shows the PL spectra of the NCs with and without ligands (URSOA-WL and URSOA-WOL) where the PL peaks for both NCs are observed at almost the same wavelengths of 523 and 525 nm, respectively. The NCs with ligands (URSOA-WL) exhibit a stronger PL intensity than the NCs without ligands (URSOA-WOL). Based on the XPS results described above, defect species are not observed at the NC surface in the case of the NCs with ligands (URSOA-WL). Under these circumstances, the radiative recombination could be more predominant than the non-radiative recombination, leading to intense PL intensity. In contrast, in the case of the NCs without ligands (URSOA-WOL), the NCs could form the surface defect species which may form the trap states within the band gap.^86^ The excitons trapped at the surface defect species could exhibit non-radiative recombination process. Thus, weaker PL intensity observed for the NCs without ligands (URSOA-WOL) may be caused by the surface defect species which might act as non-radiative decay center.

The PL decays observed in the NCs prepared by the URSOA method (URSOA-WL and URSOA-WOL) show almost similar decay characteristics, as seen in Fig. 16(b). These PL decays were fitted using a bi-exponential function with two distinct decay time constants, including their average decay time.^70,71^ The PL decays in Fig. 16(b) and the fitting results (Table 3) indicate that the NCs prepared by the URSOA method (URSOA-WL and URSOA-WOL) exhibit indistinguishable decay features. This fact implies that the excitons in these NCs might not be affected by the ligands and the surface defect species. According to the previous study, due to the zero-dimensional (0D) framework of Cs_4_PbBr_6_ crystal, strong exciton confinement might occur in the isolated individual [PbBr_6_]^4−^ octahedron.^17^ Although the NCs prepared by the URSOA method contain Cs_4_PbBr_6_ and CsPbBr_3_ phases with the ratio of nearly equals to 10 : 1, the PL might be predominantly occurred on the Cs_4_PbBr_6_ NCs. The contribution from CsPbBr_3_ NCs in the PL spectra is not seen because the PL peak of CsPbBr_3_ NCs is slightly red-shifted to a wavelength longer than 525 nm, as described in 3.1.2. The PL characteristics of these URSOA NCs indicate a higher exciton confinement in the Cs_4_PbBr_6_ NCs phase compared to the CsPbBr_3_ NCs phase.^18,87^ Regarding the possibility of phase ratio changes, it should be noted that such phase change can be disregarded by considering the XRD data of those URSOA NCs, which show the similar phase composition (hexagonal Cs_4_PbBr_6_ and orthorhombic CsPbBr_3_) in URSOA NCs with and without ligands (URSOA-WL and -WOL NCs). Therefore, a slight increase in the PL intensity of the URSOA-WL is originated from the surface ligands, which act as passivation molecules.

**Table 3:** The fitting results obtained from the curve fittings of PL decays in Fig. 16(b) using a bi-exponential function. *I*_1_ and *I*_2_ are the normalized initial intensities

Sample	*I*_1_	*τ*_1_ (ns)	*I*_2_	*τ*_2_ (ns)	*τ*_avg_ (ns)
NCs with ligand (URSOA-WL)	0.55	1.22	0.43	7.26	6.19
NCs without ligand (URSOA-WOL)	0.55	1.06	0.42	7.23	6.14

### 3.3. Comparison between NCs obtained from LARP and URSOA synthesis methods

First of all, we discuss the influence of synthesis methods on the crystal structures of the resulting NCs. Although both LARP and URSOA methods incorporate similar synthesis steps and precursor compositions, the resulting NCs have different crystal structures. The LARP method yielded orthorhombic CsPbBr_3_ NCs. However, the URSOA method yielded a mixture of the orthorhombic CsPbBr_3_ phase and the hexagonal Cs_4_PbBr_6_ NCs phase, where the phase molar ratio of Cs_4_PbBr_6_ : CsPbBr_3_ is nearly equal to 10 : 1. The LARP method facilitates a gradual crystallization process, resulting in the high crystal symmetry CsPbBr_3_ NCs. In contrast, in the URSOA method, ultrasonic waves may lead to cavitation effects and localized heating, characterized by intense atomic vibrations. This condition may hinder the formation of highly symmetric crystal structures. Under these conditions, ultrasonication tends to produce Cs_4_PbBr_6_ NCs more preferentially rather than CsPbBr_3_ NCs.^29^

Next, we discuss the relationship between the ligand and the surface chemical states. In the LARP method, LA could dissociate into a linoleate anion (L^−^) and proton (H^+^), in which the L^−^ anion tends to act as Lewis acids. During the crystal (surface) formation, the L^−^ anion might bond to the Cs^+^ and Pb^2+^ cations by using carboxyl –COO^−^ group whereas H^+^ may bond to the Br atom (as shown in Fig. 8).^52,62,88^ Therefore, the LA molecules might prevent the formation of defect states at the NC surface. In the absence of the LA ligand, since the Cs, Pb, and Br atoms at the NC surface might be unstable, these atoms could be easily removed from their sites in the crystalline structure, forming the accumulations of Cs^+^, Pb^0^, unbonded Br atoms, and Br vacancies at the NC surface. In the URSOA method, on the other hand, two kinds of ligands, namely LA and OlAm were used. The LA molecules might dissociate into L^−^ and H^+^, where L^−^ could bond to the Cs^+^ and Pb^2+^ cations and H^+^ bond to Br^−^ anions at the NC surface. In addition, H^+^ might interact with NH_2_ species of the OlAm molecules, forming OlAm^+^ or –NH_3_^+^ cations.^14,88^ These cations could bond to Br^−^ anions at the NC surface.^89^ Several previous studies have highlighted the significance of the interaction between Br^−^ and ligands, particularly the formation of Br–oleylammonium on the NC surface.^90,91^ This interaction facilitates robust passivation, thereby preventing the formation of Br vacancies and under-coordinated Pb^2+^.^90,91^ Thus, the LA and the OlAm ligands could neutralize the surface defect species, including defect vacancies, and passivate the surface defect states that could act as non-radiative recombination centers. In contrast to NCs without those ligands, where the surface defect species, such as the accumulations of Cs^+^, Pb^0^, unbonded Br atoms, and Br vacancies, were formed at the NC surface, the presence of ligands evidently prevents the formation of those surface defect species.

Finally, we also need to discuss the difference in PL characteristics observed in the NCs prepared by the LARP and URSOA methods in relation to their surface chemical states. The PL of LARP NCs mainly appears weaker than the PL of URSOA NCs. In the LARP method, we did not use OlAm ligand during the synthesis. In the URSOA method, however, OlAm ligand was used in addition to LA ligand. It has been reported the formation of Br–oleylammonium provides strong passivation and can stabilize excitons on the NC surface, which thus results in high PL efficiency.^90–92^ The presence of only Cs–oleate cannot provide effective passivation, resulting in a large number of trap states and reducing PL efficiency.^90^ Therefore, these facts strongly emphasize the effect of ligands on PL characteristics by minimizing the non-radiative recombination sites on the surface of NCs.

In addition, it is also important to consider also the difference of PL characteristics in those LARP and URSOA in relation to their crystal structures. The PL characteristics of the LARP CsPbBr_3_ NCs will be related to its orthorhombic crystal structure, where the [PbBr_6_]^4−^ octahedrons are continuously connected through a corner-sharing arrangement to form a 3D framework,^74^ as illustrated in Fig. 10(b). This 3D framework facilitates the formation of excitons with large Bohr radius and long diffusion length.^93,94^ Certain excitons may diffuse towards the surfaces of the NCs, where they subsequently emit PL. However, certain excitons may be trapped at the surface defect sites, which can undergo nonradiative recombination or emit PL at a lower photon energy and intensity. Consequently, PL quenching may occur and shorten the PL lifetime, as observed in the NCs without ligand (LARP-WOL). Different situation occurs in the URSOA NCs consisting of the predominant Cs_4_PbBr_6_ NCs structure, where each [PbBr_6_]^4−^ octahedron is surrounded by the Cs atoms being isolated without sharing the corner atoms with neighboring [PbBr_6_]^4−^ octahedron (Fig. 10(c)). In this arrangement, [PbBr_6_]^4−^ octahedron could be rather isolated, forming 0D arrangement.^28,87^ This 0D arrangement could confine the excitons within the individual octahedra, showing the excitons with a smaller Bohr radius.^87,95^ As a result, the peak position does not change and PL intensity is just slightly decreased.

## 4. Conclusion

Cesium lead bromide perovskite NCs have been synthesized using the LARP and URSOA methods, where those methods used similar processing steps and precursor solutions. However, the crystal structures prepared by these methods were significantly different from each other. The LARP method yielded the CsPbBr_3_ NCs with orthorhombic crystal structures, whereas the URSOA method yielded a mixture of hexagonal Cs_4_PbBr_6_ and orthorhombic CsPbBr_3_ NCs. From HAXPES, it was found that the chemical states of the interior or inner side of the NCs were not affected by the presence or absence of the ligands. On the other hand, from XPS, the NCs without the ligand showed additional chemical states originating from the accumulation of Cs cations, Pb^0^, unbonded Br atoms, and Br vacancies at the NCs surface. These surface chemical states can be then associated with surface defects, which can act as non-radiative recombination sites. The PL of the LARP (orthorhombic CsPbBr_3_) NCs exhibits two distinct PL decay components attributed to free and trapped excitons. This structure, characterized by its 3D crystal framework, may facilitate exciton diffusion or migration to the surface of the NCs. Therefore, excitons reaching the NCs without ligands may undergo non-radiative recombination, resulting in a weak PL with a short PL lifetime. Here, excitons in CsPbBr_3_ NCs are sensitive to surface states or surface defects. In contrast, despite the PL characteristics of the URSOA NCs also show two PL decay components, also being associated with free and trapped excitons, the PL characteristics of both NCs with and without ligands are not really sensitive to the presence or absence of ligands. This characteristic may arise from the 0D crystal framework of the Cs_4_PbBr_6_ NCs, where excitons are more localized in the [PbBr_6_]^4−^ octahedron without long migration or diffusion to the NCs surface. Therefore, URSOA NCs are more tolerant to the presence of surface defects compared to LARP NCs. The present experimental findings in this study may provide new insights into the effect of ligands on the NCs surface structures, which are associated with the formation of additional surface chemical states originating from surface defect species, and their impact on PL characteristics of these lead-halide perovskite materials. It may be useful not only for further development of passivation molecules for halide perovskites in general but also in developing buffer layer molecules for perovskite heterojunction devices.

## Conflicts of interest

There are no conflicts of interest to declare.

## Supplementary Material

RA-015-D5RA05099E-s001

## Data Availability

The data that supports the findings of this study are available from the corresponding authors upon reasonable request. The individual XPS survey spectra, the fitting parameters of the HAXPES and XPS analyses, and the FTIR assignments. These data were used for the results presented in the main manuscript. See DOI: https://doi.org/10.1039/d5ra05099e.
