# Supplementary material for: The effect of surface ligands on the surface chemical states and photoluminescence characteristics in cesium lead bromide perovskite nanocrystals
Source: RSC Adv. 2025 Aug 28;15(37):30727–41. doi: 10.1039/d5ra05099e (PMC12395022; doi:10.1039/d5ra05099e)
Supplement: RA-015-D5RA05099E-s001 [file RA-015-D5RA05099E-s001.pdf]

Supplementary Information (SI) for RSC Advances.
This journal is © The Royal Society of Chemistry 2025

Supplementary informations of "The Effect of Surface Ligands on the Surface Chemical States and Photoluminescence Characteristics in Cesium Lead Bromide Perovskite Nanocrystals"

Muhammad Asharuddin[a], Rahmat Hidayat[a*], Adhita Asma Nurunnizar[b], Natalita Maulani Nursam[b], Valdi Rizki Yandri[c], Waode Sukmawati Arsyad[d], Joko Suwardy[e], Efi Dwi Indari[e], and Yoshiyuki Yamashita[f,g*]

[a] Physics of Magnetism and Photonics Research Division, Faculty of Mathematics and Natural Sciences, Bandung Institute of Technology, Jl. Ganesha 10, Bandung 40132, West Java, Indonesia
[b] Research Center for Electronics, National Research and Innovation Agency (BRIN), KST Samaun Samadikun, Jl. Sangkuriang, Bandung 40135, West Java, Indonesia
[c] Department of Electrical Engineering, Polytechnic State of Padang, Limau Manis Padang 25164, West Sumatra, Indonesia
[d] Physics Department, Faculty of Mathematics and Natural Sciences, Halu Oleo University, Anduonohu, Kendari, South East Sulawesi, 93232, Indonesia
[e] Research Center for Quantum Physics, National Research and Innovation Agency (BRIN), KST BJ Habibie Serpong, Banten, Indonesia 15314
[f] Nano Electronics Device Materials Group, Research Center for Electronic and Optical Materials, National Institute for Materials Science (NIMS), 305-0044 1-1 Namiki Tsukuba Ibaraki, Japan
[g] Graduate School of Engineering, Kyushu University, Motooka 744, Nishi-ku, Fukuoka 819-0395, Japan

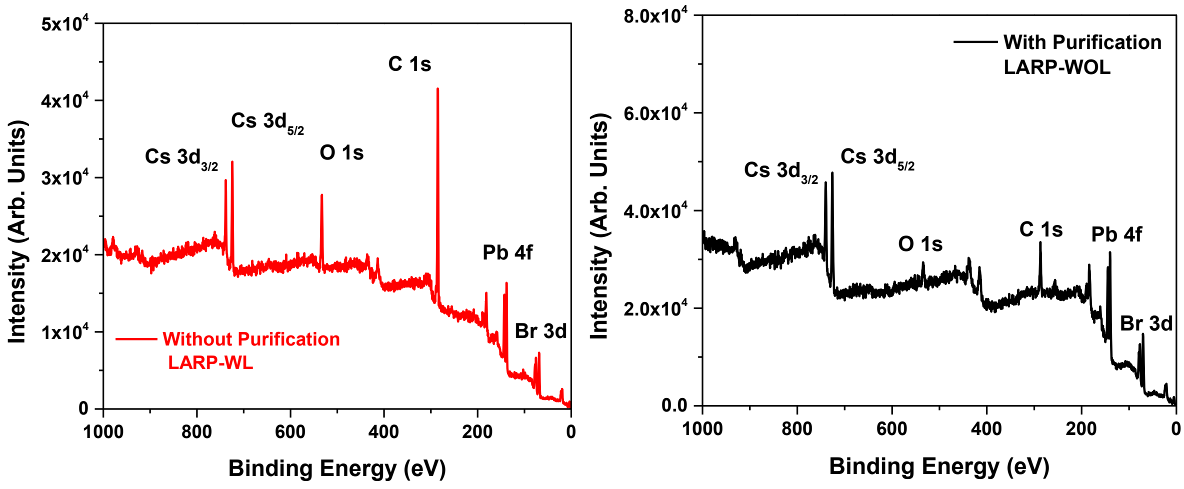

Fig. S1. The XPS survey spectra measured from LARP NCs without and with purification (LARP-WL NCs and LARP-WOL NCs)

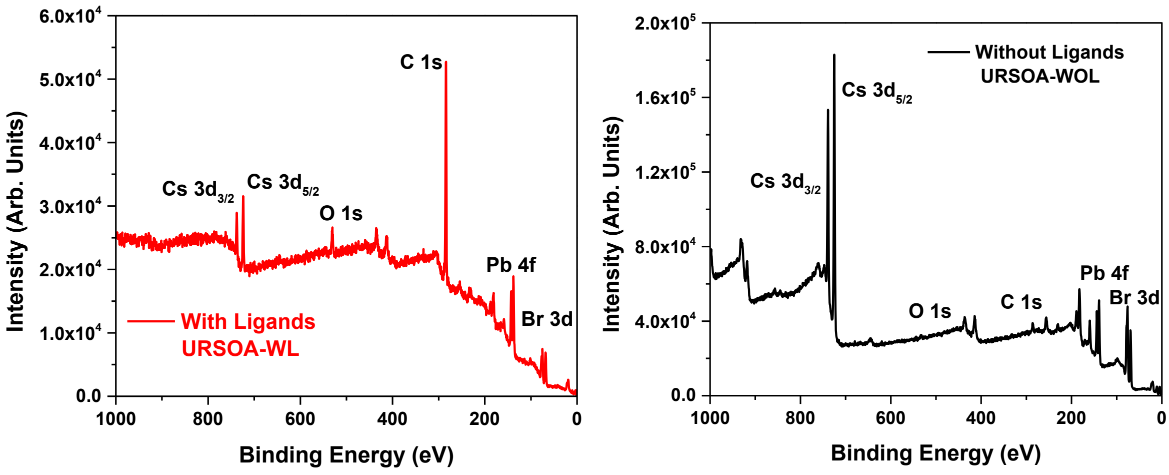

Fig. S2. The XPS survey spectra measured from URSOA NCs with and without ligands (URSOA-WL NCs and URSOA-WOL NCs)

Table S.1. HAXPES parameters fitting of LARP-WL and LARP-WOL samples (Figure 4).

Sample name	Core Level	Position (eV)	Assigned State	Lorentzian width	Gaussian width	Area
LARP-WL	Cs $3d_{5/2}$	724.2	Cs-$CsPbBr_3$	0.80	1.03	172.9
	Pb 4f	138.2	Pb-$CsPbBr_3$	0.22	1.04	167.0
	Br 3d	68.2	Br-$CsPbBr_3$	0.10	1.10	33.1
LARP-WOL	Cs $3d_{5/2}$	724.2	Cs-$CsPbBr_3$	0.85	0.95	498.6
	Pb 4f	722.9	Pb-$CsPbBr_3$	0.46	0.92	296.0
	Br 3d	68.2	Br-$CsPbBr_3$	0.37	1.00	99.8

Table S.2. XPS parameters fitting of LARP-WL and LARP-WOL samples (Figure 6).

Sample name	Core Level	Peak position (eV)	Assigned State	Lorentzian width	Gaussian width	Area	%
LARP-WL	Cs $3d_{5/2}$	724.2	Cs-$CsPbBr_3$	0.68	0.95	7,137	100.0
	Pb 4f	138.2	Pb-$CsPbBr_3$	0.31	0.77	7,222	100.0
	Br 3d	68.2	Br-$CsPbBr_3$	0.23	0.84	3,333	100.0
LARP-WOL	Cs $3d_{5/2}$	724.2	Cs-$CsPbBr_3$	0.46	1.45	17,544	78.9
	Cs $3d_{5/2}$	722.9	Cs-accumulation	1.23	1.16	4,686	21.1
	Pb 4f	138.0	Pb-$CsPbBr_3$	0.07	1.29	10,837	76.5
	Pb 4f	136.8	Pb^0	0.33	1.53	3,323	23.5
	Br 3d	68.0	Br-$CsPbBr_3$	0.40	1.13	4,786	82.1
	Br 3d	66.8	Unbonded Br	1.14	0.82	1,040	17.9

Table S.3. Assignments of chemical group vibrations of FTIR spectra of LARP-WL and LARP-WOL samples (Figure 7).

LARP Without Purification (LARP-WL)			With Purification LARP-WOL			References
Wavenumber (cm^{-1})	Intensity*	Assignments	Wavenumber (cm^{-1})	Intensity*	Assignments	
720.0	m	C–C bending				[1]
957.0	w	C–H bending				[2]
1,156.0	m	C–O stretching				[2]
1,453.8	m	COO^- stretching symmetrical	1450.0	vw	COO^- stretching symmetrical	[3,4]
1,712.9	s	C=O stretching	1710.5	vw	C=O stretching	[2,3,5,6]
2,850.0	s	C-H_2 stretching	2848.6	vw	C-H stretching	[2,3,5,6]
2924.0	s	C-H_2 stretching	2921.6	vw	C-H stretching	[2,3,5,6]
3012.0	vw	C-H-C=C stretching				[2,3,5,6]

* s : strong, m: medium, w: weak, vw: very weak

Table S.4. HAXPES parameters fitting of URSOA-WL and URSOA-WOL samples (Figure 11).

Sample name	Core Level	Peak position (eV)	Assigned State	Lorentzian width	Gaussian width	Area
URSOA-WL	Cs $3d_{5/2}$	724.0	Cs-$CsPbBr_3$	1.60	0.50	174.8
	Cs $3d_{5/2}$	723.3	Cs-Cs_4PbBr_6	0.83	0.97	1748.5
	Pb 4f	138.0	Pb-$CsPbBr_3$	0.50	0.80	18.7
	Pb 4f	137.4	Pb- Cs_4PbBr_6	0.60	0.88	186.6
	Br 3d	68.2	Br-$CsPbBr_3$	0.38	0.60	10.3
	Br 3d	67.2	Br-Cs_4PbBr_6	0.12	1.12	102.7
URSOA-WOL	Cs $3d_{5/2}$	724.0	Cs-$CsPbBr_3$	1.59	0.50	222.1
	Cs $3d_{5/2}$	723.4	Cs-Cs_4PbBr_6	0.61	1.07	2220.7
	Pb 4f	138.0	Pb-$CsPbBr_3$	0.50	0.81	20.1
	Pb 4f	137.4	Pb- Cs_4PbBr_6	0.34	0.96	201.1
	Br 3d	68.2	Br-$CsPbBr_3$	0.50	0.63	15.2
	Br 3d	67.2	Br-Cs_4PbBr_6	0.31	0.97	151.9

Table S.5. XPS parameters fitting of URSOA-WL and URSOA-WOL samples (Figure 13).

Sample name	Core Level	Peak position (eV)	Assigned State	Lorentzian width	Gaussian width	Area	%
URSOA-WL	Cs $3d_{5/2}$	724.0	Cs-$CsPbBr_3$	0.50	0.87	686	9.1
	Cs $3d_{5/2}$	723.3	Cs-Cs_4PbBr_6	0.86	1.04	6,856	90.9
	Pb 4f	138.0	Pb-$CsPbBr_3$	0.50	0.49	576	9.1
	Pb 4f	137.3	Pb- Cs_4PbBr_6	0.47	1.00	5,760	90.9
	Br 3d	68.0	Br-$CsPbBr_3$	0.60	0.70	222	9.1
	Br 3d	67.2	Br-Cs_4PbBr_6	0.50	0.99	2,222	90.9
URSOA-WOL	Cs $3d_{5/2}$	724.0	Cs-$CsPbBr_3$	0.23	1.22	11,276	8.7
	Cs $3d_{5/2}$	723.3	Cs-Cs_4PbBr_6	1.08	1.11	112,759	86.7
	Cs $3d_{5/2}$	722.0	Cs-accumulation	0.34	1.00	6,068	4.7
	Pb 4f	138.0	Pb-$CsPbBr_3$	0.50	1.31	1,948	8.3
	Pb 4f	137.4	Pb- Cs_4PbBr_6	0.20	0.73	19,480	83.2
	Pb 4f	136.2	Pb^0	0.24	1.00	2,000	8.5
	Br 3d	68.0	Br-$CsPbBr_3$	0.69	0.70	1,451	8.5
	Br 3d	67.2	Br-Cs_4PbBr_6	0.88	0.80	14,518	84.9
	Br 3d	66.7	Unbonded Br	0.33	0.68	1,138	6.7

Table S.6. Assignments of chemical group vibrations of FTIR spectra of URSOA-WL and URSOA-WOL samples (Figure 14).

URSOA With Ligand (URSOA-WL)			URSOA Without Ligand (URSOA-WOL)			References
Wavenumber (cm^{-1})	Inten-sity*	Assignments	Wavenumber (cm^{-1})	Inten-sity*	Assignments	
723	m	C–C bending vibration				[1]
947	vw	C–H bending vibration				[2]
1,019	vw	C-N stretching				[7]
1,400	m	COO^- stretching symmetrical				[6]
1,464	vw	C-H Scissoring				[2]
1,538	m	COO^- stretching asymmetrical or N-H banding				[3,5,6]
1,731	vw	C=O streching				[6]
2,849	s	C-H_2 stretching	2846	vw	C-H streching	[2,3]
2,919	s	C-H_2 stretching	2920	vw	C-H streching	[2,3]
3,006	vw	C-H-C=C streching				[2]

* s : strong, m: medium, w: weak, vw: very weak

References

[1] M. R. Patel, T. J. Park, and S. K. Kailasa, Synthesis of Green Fluorescence CsPbBr3 Perovskite Quantum Dots Via Probe Sonication for the Detection of Isoprothiolane Fungicide in Food Samples, J Clust Sci **35**, 2105 (2024).

[2] B. M. Abdullah, N. Salih, and J. Salimon, Optimization of the chemoenzymatic mono-epoxidation of linoleic acid using D-optimal design, Journal of Saudi Chemical Society **18**, 276 (2014).

[3] I. O. Perez De Berti, M. V. Cagnoli, G. Pecchi, J. L. Alessandrini, S. J. Stewart, J. F. Bengoa, and S. G. Marchetti, Alternative low-cost approach to the synthesis of magnetic iron oxide nanoparticles by thermal decomposition of organic precursors, Nanotechnology **24**, 175601 (2013).

[4] Y. Lu and J. D. Miller, Carboxyl Stretching Vibrations of Spontaneously Adsorbed and LB-Transferred Calcium Carboxylates as Determined by FTIR Internal Reflection Spectroscopy, Journal of Colloid and Interface Science **256**, 41 (2002).

[5] S. Akhil, V. G. V. Dutt, and N. Mishra, Surface modification for improving the photoredox activity of $CsPbBr_3$ nanocrystals, Nanoscale Adv. **3**, 2547 (2021).

[6] I. A. Shuklov, V. F. Toknova, D. V. Demkin, G. I. Lapushkin, L. M. Nikolenko, A. A. Lizunova, S. B. Brichkin, V. N. Vasilets, and V. F. Razumov, A New Approach to the Synthesis of Lead Sulfide Colloidal Quantum Dots in a Mixture of Oleylamine and Oleic Acid, **54**, (2020).

[7] Z. Fereshteh, M. Salavati-Niasari, K. Saberyan, S. M. Hosseinpour-Mashkani, and F. Tavakoli, Synthesis of Nickel Oxide Nanoparticles from Thermal Decomposition of a New Precursor, J Clust Sci **23**, 577 (2012).
